# The significance of perioperative glucocorticoids in the prevention of seroma formation after mastectomies: a systematic review and meta-analysis

**DOI:** 10.1007/s10549-025-07830-3

**Published:** 2025-11-25

**Authors:** Levente Doleviczényi, Lőrinc Frivaldszky, Anett Rancz, Dóra Léna Fedorcsák, Boglárka Lilla Szentes, Péter Hegyi, Zoltán Klárik

**Affiliations:** 1 Centre for Translational Medicine, Semmelweis University, 1085 Baross Utca 22., Budapest, Hungary; 2 Department of Surgery, Transplantation and Gastroenterology, Semmelweis University, 1082 Üllői Út 78., Budapest, Hungary; 3 Department of Obstetrics and Gynecology, Semmelweis University, 1082 Üllői Út 78., Budapest, Hungary; 4 Institute of Pancreatic Diseases, Semmelweis University, 1082 Üllői Út 78., Budapest, Hungary; 5 MRE Bethesda Children’s Hospital, 1146 Bethesda Utca 3., Budapest, Hungary; 6 Institute for Translational Medicine, Medical School, University of Pécs, 7624 Szigeti Út 12., Pécs, Hungary

**Keywords:** Breast cancer, Mastectomy, Seroma, Glucocorticoid, Methylprednisolone, Hydrocortisone

## Abstract

**Purpose:**

Seroma formation is one of the most common complications after mastectomy. Seromas can lead to repeated aspirations, increase the risk of infection, and potentially delay oncologic treatment. Several strategies have recently been employed to prevent seromas, but there is no definitive standard. We aim to determine whether perioperative glucocorticoids (GC) are safe and effective in preventing seromas in patients undergoing mastectomy.

**Methods:**

We performed a systematic search in five databases on November 12, 2024. Eligible studies included women who underwent mastectomy and received perioperative GCs. Results are reported as risk ratios (RR), odds ratios (OR), or mean differences (MDs) with 95% confidence intervals (CIs), and are presented as forest plots. A random-effects model was used to pool effect sizes.

**Results:**

Altogether, 13 studies (12 RCTs and 1 case–control study) with 1011 patients were included; all were eligible for meta-analysis. The rate of seroma formation was significantly lower in the GC groups compared to the placebo groups (RR = 0.56, CI 0.38; 0.82, p = 0,008). Total volume of drainage (MD = -213.36 ml, CI -312.5; -114.22, p = 0.001) and days to drain removal (MD =—3.01 days, CI -4.06; -1.96, p = 0.001) were also lower in the GC groups. The rate of wound infection showed a higher trend in the intervention groups (RR = 1.26, CI 0.82; 1.92, p = 0.224), although the results did not reach statistical significance,

**Conclusions:**

Our results suggest that perioperative glucocorticoid administration may reduce seroma formation in patients undergoing mastectomy. A potential increase in wound infection rates was also observed, but this requires further investigation.

**Supplementary Information:**

The online version contains supplementary material available at 10.1007/s10549-025-07830-3.

## Introduction

Breast cancer is one of the most common cancers in women, with over 2,2 million cases diagnosed worldwide in 2022 [1]. Optimal disease management requires a multidisciplinary approach in which surgery still serves as a main pillar. Treatment options include breast-conserving surgeries and mastectomies with either sentinel lymph node dissection (SLND) or axillary dissection (AD). Seroma formation is the most common postoperative complication after mastectomies; literature reports it can occur in 3–85% of cases [2, 3].

Seroma, by definition, is a palpable collection of serous content accumulated in the operative area, which is currently believed to be a combination of lymphatic fluid and exudate due to an inflammatory reaction [4]. It can lead to patient discomfort, prolonged hospital stays, multiple aspirations, and wound complications such as wound infection—thus, it can even delay adjuvant treatment [5] and deteriorate quality of life, too. Risk factors are not fully explored; they include the use of electrocautery during dissection [6] and the patient's high weight [4] and body mass index (BMI) [7]. Other potential factors could be age, diabetes mellitus, hypertension, the extent of surgical procedures, and poor patient health status [8].

Although drain-free approaches have been tried before [9], closed suction drainage after mastectomies is still a well-established method recommended by guidelines. In recent years, many strategies have been used to prevent seroma formation, such as perioperative glucocorticoid (GC) administration, quilting sutures [10, 11], tranexamic acid use [12, 13], fibrin glues [14, 15], gentamicin–collagen sponges [16], or octreotide use [17]. Currently, there is no consensus on which technique is the most effective.

GCs are widely used in various diseases due to their anti-inflammatory attributes. They attenuate the surgery-induced local and systemic inflammatory reactions [18] in the surgical setting. GCs are widely used in oral and maxillofacial surgery, reducing postoperative morbidities, such as pain and edema [19]. Perioperative GC use was observed to improve myocardial function after cardiac surgery [20] and lower seroma re-accumulation rates were demonstrated after latissimus dorsi flap harvest [21]. The meta-analysis by Srinivasa et al. [22] assessed the effect of preoperative GCs, mainly in colorectal and liver surgeries, finding lower total complication rates in the intervention groups.

On the other hand, perioperative GC use could lead to complications, such as wound infection [23], hyperglycemia [24] or impaired wound healing [24]. Although many GC-related adverse effects occur after high-dose or chronic use, careful management and risk–benefit assessment are crucial when using perioperative GCs.

In breast surgery, GCs are given either as a preoperative (intravenous) or as a postoperative (intracavitary) method. Many individual studies demonstrated promising results, but there is no comprehensive data on this topic yet. We aimed to investigate the efficacy and safety of perioperative GC use in reducing seroma formation in patients undergoing mastectomies.

## Methods

We report our systematic review and meta-analysis based on the recommendation of the PRISMA [25] (Preferred Reporting Items for Systematic Reviews and Meta-Analyses) 2020 guideline (see Supplementary Table 1) and the Cochrane Handbook [26]. The study protocol was registered on PROSPERO (International Prospective Register of Systematic Reviews) with the registration number CRD42024612867 [27], and we wholly adhered to it.

## Literature search and eligibility criteria

Our systematic literature search was conducted on the 12th of November 2024 in 5 databases—MEDLINE (via PubMed), Embase, CENTRAL (Cochrane Central Register of Controlled Trials), Scopus, and Web of Science. The detailed search strategy—including the number of hits in each database—can be found in the Supplementary Material (Table S2). Citation chasing and manual reference checking were performed on the 12th of December, 2024.

We included both randomized and observational comparative studies in our analysis reporting on female patients undergoing mastectomies due to breast cancer treatment (population) and receiving perioperative GCs (intervention) if they provided data on the outcomes of interest (outcome). Control groups used placebo or blank control (comparator). Case reports and series, where no full-text available and studies involving patients having GC allergy or receiving chronic GC treatment were excluded from our analysis.

Our primary outcomes were seroma formation and wound infection rates. Seroma was defined as a palpable mass of serous fluid requiring aspiration. Wound infection was defined as a surgical site infection with or without the need of antibiotic treatment. Secondary outcomes included further wound complications (wound necrosis and dehiscence) and outcomes regarding drainage (total volume of drainage, days to drain removal).

## Study selection and data collection process

After the duplicates were removed using EndNote 20 (Clarivate Analytics, Philadelphia, PA, USA), the selection was performed using the Rayyan Systems software tool (Cambridge, MA, USA) by two independent review authors (L.D. and D.L.F.). We reviewed the publications by title, abstract, and full text. Disagreements were resolved by a third author (Z.K.).

Two authors (L.D. and D.L.F.) independently collected data from the eligible articles into previously defined Excel sheets (Microsoft, Redmond, WA, USA). The following data were extracted: first author, the year of publications, DOI (Digital Object Identifier) or PMID (PubMed Identifier), study population, study period, study site, study design, age, BMI, type of surgery, dissection method, number of drains, type of GC used, dosage, timing of intervention (pre- or postoperative) and any relevant data on primary and secondary outcomes.

## Risk of bias and quality of evidence assessment

Two authors (L.D. and D.L.F.) performed the risk of bias assessment independently using the revised Cochrane Risk of Bias 2 (RoB 2) [28] tool for the included RCTs and the Cochrane Risk of Bias in Non-randomized Studies of Interventions (ROBINS-I) [29] for the eligible nonrandomized studies. Disagreements were resolved by the third author (Z.K.). The quality of evidence was assessed according to the recommendations of the Grades of Recommendation, Assessment, Development, and Evaluation (GRADE) workgroup [30].

## Synthesis methods

Meta-analyses were performed using random-effects models throughout, given the expectation of between-study heterogeneity. For dichotomous outcomes, we pooled risk ratios (RR) with 95% confidence intervals (CI) when including only randomized controlled trials, and odds ratios (OR) when observational studies were analyzed. For continuous outcomes, mean differences (MD) with 95% CI were computed by subtracting the mean of controls from that of experimental groups.

Effect sizes were pooled using established methods for binary and continuous data: the Mantel–Haenszel method was used for RR and OR, and the inverse variance method for MD [31]. Heterogeneity was assessed through the I2 statistic and estimation of between-study variance (τ2) using the Paule–Mandel or restricted maximum-likelihood estimators, as appropriate [32–35]. When feasible, prediction intervals were reported. Publication bias was evaluated by funnel plots and Peters’ test, and outlier or influential studies were identified using influence analyses [33, 36].

All analyses were conducted in R (v4.4.2) with the meta and dmetar packages [37, 38].

## Results

### Search and selection

**Fig. 1:**
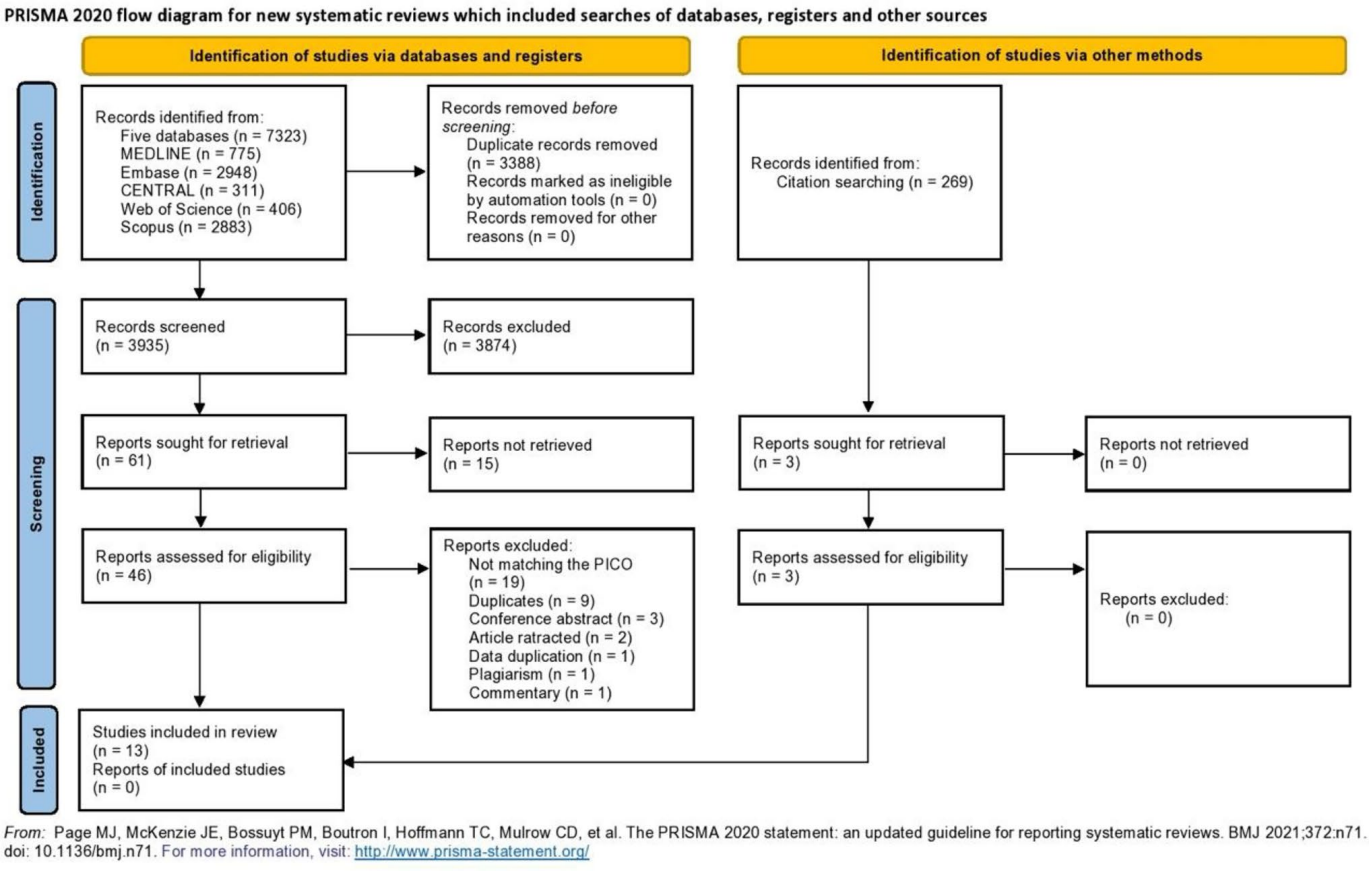
PRISMA flowchart for search and selection

Altogether, 7323 studies were identified by our systematic search. After full-text selection, citation searching [39] and manual reference checking, 13 articles [40–52] were included. The search and selection process is shown in Fig. 1.

## Basic characteristics of included studies

Twelve RCTs and one case–control study were included, published between 2011 and 2024. One study [47] self-identified as a case–control study, but based on the manuscript, the design is of an RCT, as highlighted by Aschraf et al. [53]. The baseline characteristics of the enrolled articles are detailed in Table 1. In Supplementary Material Table S3, we listed additional information on the studies; in Table S4, we summarized the inclusion and exclusion criteria of the included papers.

**Table 1:** Basic characteristics of included studies

Author (year)	Study site	Number of analyzed patients	Study type	Glucocorticoid use	Dosage	Timing of intervention	Type of surgery	Relevant outcomes
Albatanony et al. 2021[40]	Egypt	60	RCT	HC + TA	1 × 100 mg HC + 1 × 5 ml TA	preoperative	MRM	Seroma formation, TVD, DDR
Fatima et al. 2024[41]	Pakistan	152	RCT	HC	1 × 100 mg	preoperative	MRM	Seroma formation and aspiration, TVD, DDR
Iqbal et al. 2023[42]	Pakistan	60	RCT	MP	1 × 120 mg	preoperative	MRM	TVD
Khaleel et al. 2023[43]	India	62	RCT	MP	1 × 125 mg	preoperative	MRM	Seroma formation and aspiration, wound infection, TVD
Khan et al. 2017[44]	Pakistan	65	RCT	MP	1 × 120 mg	preoperative	Simple mastectomy, MRM, MRM + AD	Seroma formation, wound infection, dehiscence and necrosis, TVD, DDR
Okholm et al. 2011[45]	Denmark	42	RCT	MP	1 × 125 mg	preoperative	Mastectomy + AD/SLND	Seroma formation, wound infection and necrosis
Qvamme et al. 2015[46]	Denmark	212	RCT	MP	1 × 80 mg	postoperative	Mastectomy + AD/SLND	Seroma formation and aspiration, wound infection, dehiscence and necrosis
Seth et al. 2023[47]	Pakistan	60	RCT^*^	MP	1 × 120 mg	preoperative	MRM	Seroma formation, wound infection, dehiscence and necrosis, TVD, DDR
Setiawan et al. 2014[48]	Indonesia	30	RCT	MP	ND	ND	MRM	TVD
Shiraz et al. 2022[49]	Pakistan	76	RCT	MP	1 × 80 mg	postoperative	MRM	Seroma formation
Subramanian et al. 2023[50]	India	72	RCT	MP	1 × 80 mg	postoperative	Mastectomy + AD	Wound infection, TVD, DDR
Talha et al. 2014[51]	Egypt	80	RCT	HC	2 × 100 mg	preoperative	MRM	Seroma formation, TVD, DDR
Vijayalakshmi et al. 2018[52]	India	40	Case–control study	MP	1 × 125 mg	preoperative	MRM	Seroma aspiration

^*^The authors identified the study as a case–control study, but based on the report, it is designed as an RCT

AD: axillary dissection; DDR: days to drain removal; HC: hydrocortisone; MP: methylprednisolone; MRM: modified radical mastectomy; ND: not defined; RCT: randomized controlled trial; SLND: sentinel lymph node dissection; TA: tranexamic acid; TVD: total volume of drainage

## Seroma formation rate

Nine randomized controlled trials with 806 patients included data on seroma formation. Six studies used MP, while the other three studies administered HC. The timing was preoperative in seven cases and postoperative in two studies. Using GCs has significantly reduced seroma formation in the intervention groups (RR = 0,56, 95% CI 0,38; 0,82, p = 0,008). Figure S1 demonstrates an additional analysis based on the timing of the intervention.

Subgroup analysis by glucocorticoid type produced a statistically significant test for subgroup differences (p = 0.01), indicating heterogeneity in treatment effects. A nonsignificant tendency of lower seroma formation rates in the HC group (RR = 0.34, 95% CI 0.10; 01.16, p = 0.063) compared to the MP group (RR = 0.75, 95% CI 0.55; 1.04, p = 0.071) was observed. However, neither HC nor MP subgroups reached statistical significance individually, likely due to limited participant numbers. Therefore, these findings should be interpreted with caution, and further studies are needed to confirm differential effects among glucocorticoid types. The subgroup analysis by timing of intervention indicated a significant reduction in seroma formation rates with preoperative glucocorticoid administration (RR = 0.51, 95% CI 0.30; 0.86, p = 0.021), while the postoperative subgroup (RR = 0.70, 95% CI 0.09; 5.54, p = 0.272) showed a non-significant trend in the same direction. Based on the influential analysis, there were no outlier studies.

## Wound infection rate

Six studies with 500 patients reported data on wound infection rates. Preoperative steroids were used in four studies, and in two studies, GC was injected into the cavity postoperatively. All these used MP as the choice of drug. Despite not reaching statistical significance, the result suggests that wound infection rates are more common in the intervention group (RR = 1.26, 95% CI 0.82; 1.92, p = 0.224).

The subgroup analysis indicated a nonsignificant trend toward lower wound infection rates with postoperative GCs (RR = 1.12, 95% CI 0.02; 61.20, p = 0.785) compared to preoperative use (RR = 1.39, 95% CI 0.69; 2.79, p = 0.235), but these differences were not statistically meaningful. Influential analysis showed no outliers.

## Total volume of drainage

**Fig. 2:**
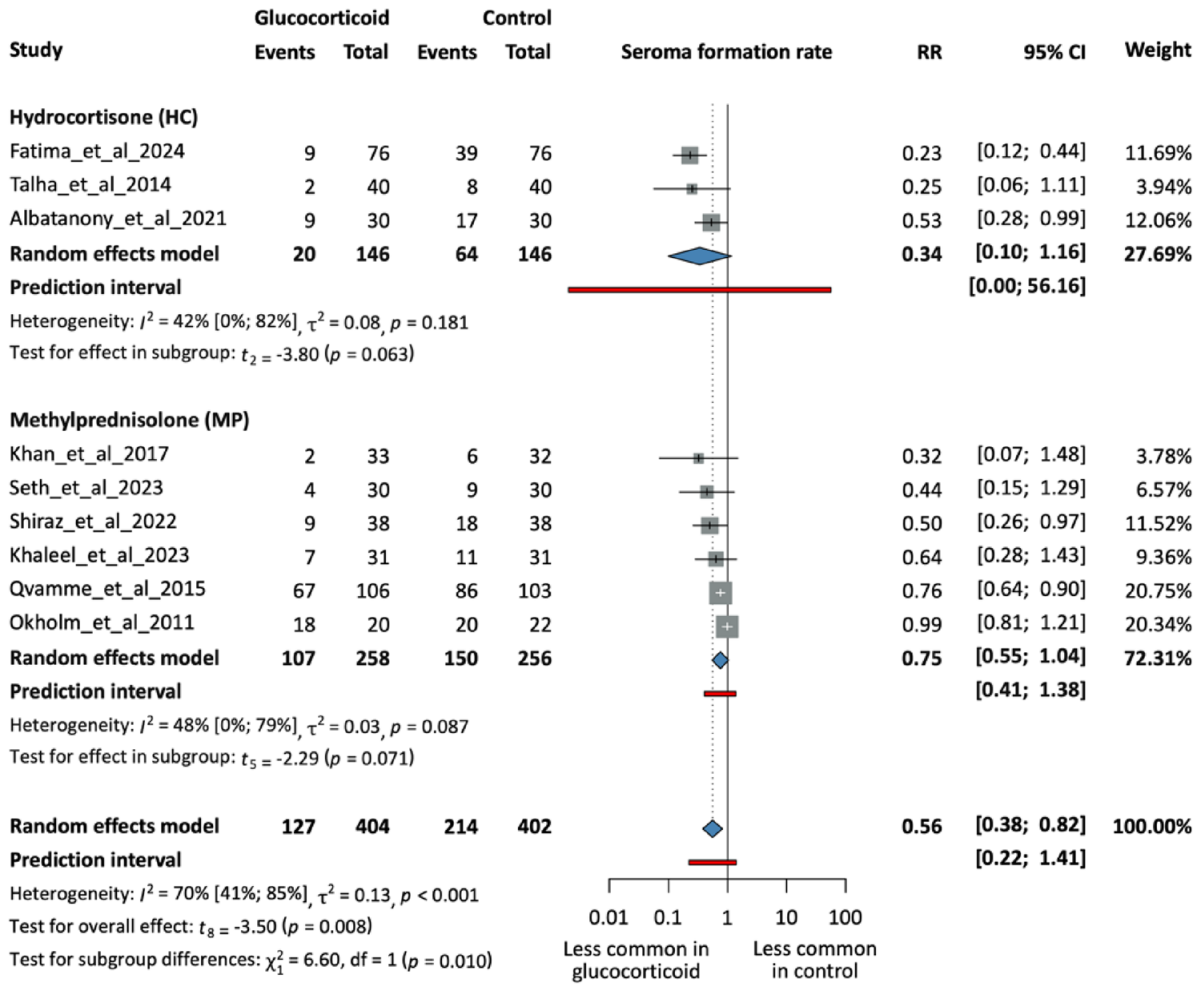
Forest plot demonstrating seroma formation rates in the intervention and control groups. Subgroups based on GC types. N, number of patients in each arm; RR, risk ratio; CI, confidence interval

**Fig. 3:**
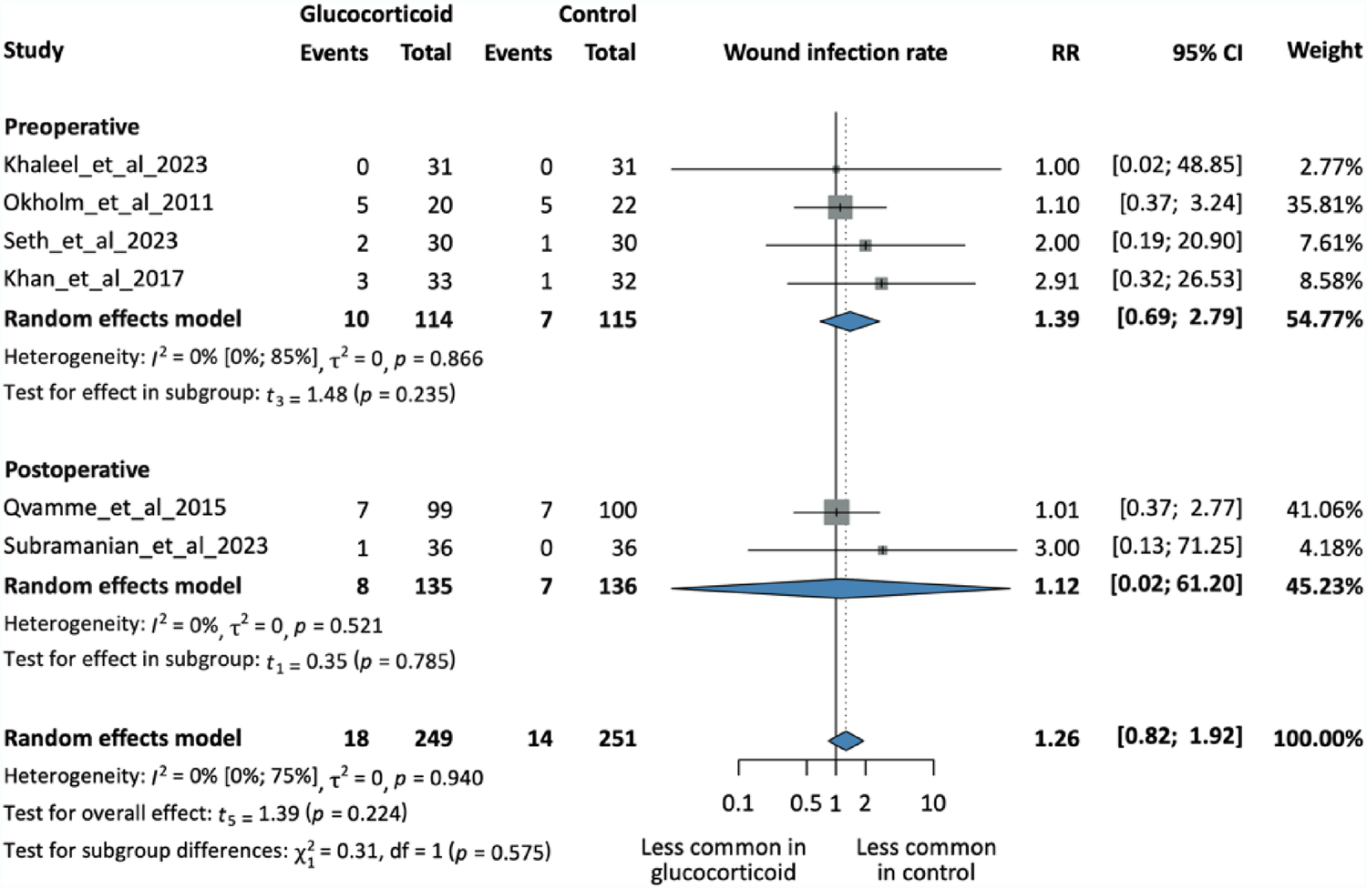
Forest plot showing wound infection rates in the intervention and control groups. Subgroups based on the timing of intervention. N, number of patients in each arm; RR, risk ratio; CI, confidence interval

**Fig. 4:**
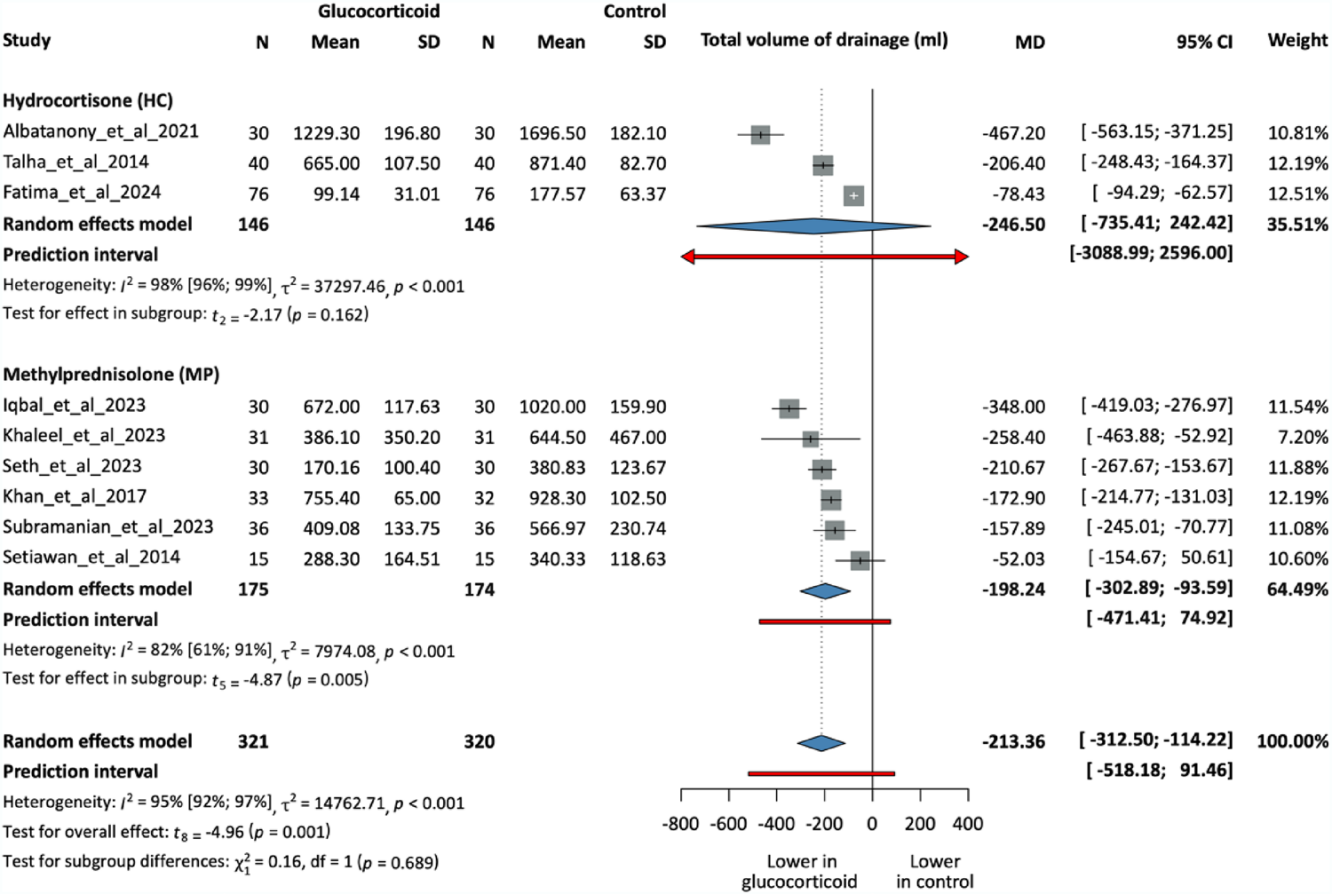
Forest plot showing the total volume of drainage in the intervention and control groups. Subgroups based on GC types. N, number of patients in each arm; MD, mean difference; CI, confidence interval

**Fig. 5:**
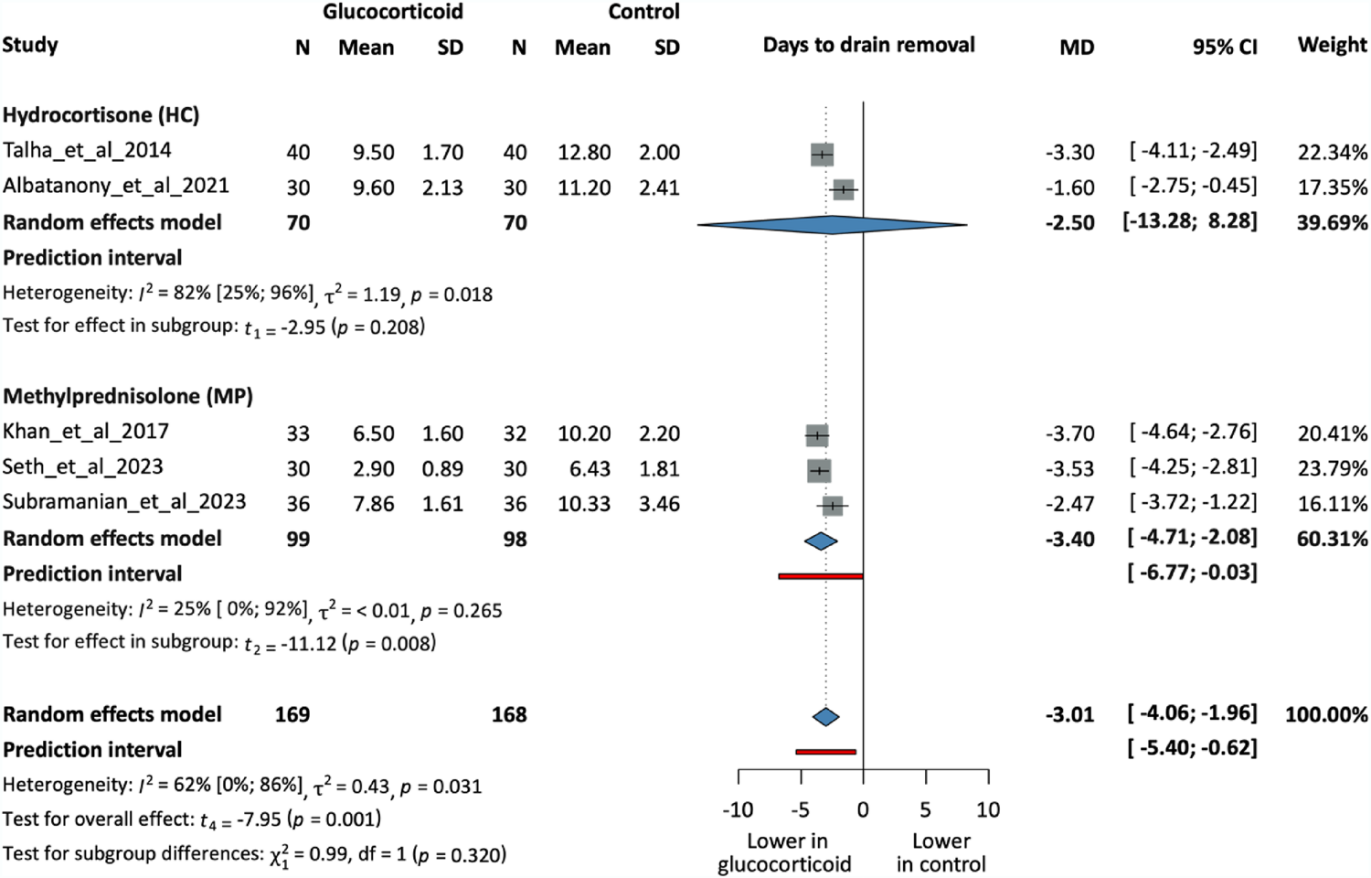
Forest plot demonstrating the length of drainage in the intervention and control groups. Subgroups based on GC types. N, number of patients in each arm; MD, mean difference; CI, confidence interval

Nine RCTs, including 641 patients, have measured the total drainage volume among the patients. All but one study reported a preoperative intervention. Six studies used MP as the choice of GC, while the other three administered HC. GC administration effectively reduced drainage volumes (MD = −213.36 ml, 95% CI −312.5; −114.22, p = 0.001), which was statistically significant. Figure S2 displays additional analysis based on the timing of the intervention (Figs. 2, 3, 4, 5).

Interpretation of the subgroup analysis between HC (MD = −246.50 ml, 95% CI −735.41; −242.42, p = 0.162) and MP groups (MD = −198.24 ml, 95% CI −302.89; −93.59, p = 0.005) is limited because of low statistical power; the results suggest a possible difference in total drainage volume, but confidence intervals are wide and heterogeneity is high, so firm conclusions cannot be drawn. Based on the influential analysis, Albatanony et al.[40] was a slight outlier, possibly due to the addition of tranexamic acid.

## Days to drain removal

Five trials with 337 patients reported data on the duration of drainage. Four studies’ approach was preoperative GC use, while one study administered GC postoperatively. Three studies used MP, and two studies chose HC as an intervention. Each of these studies used an output-based approach and removed drains when the drainage volume per 24 h was less than 30–50 ml. Drains were removed significantly earlier in the intervention groups (MD = −3.01 days, 95% CI −4.06; −1.96, p = 0.001). Figure S3 presents a subgroup analysis based on the timing of the intervention.

Subgroups could not be compared because of the low number of studies in the different groups. The influential analysis demonstrated no severe outliers.

## Further outcomes

The outcomes “seroma aspiration rate” (OR = 0.25, 95% CI 0.02; 2.77, p = 0.163), “drainage on first post-operative day” (MD = −19.26 ml, 95% CI −9391; −55.38, p = 0.382), “wound necrosis rate” (RR = 0.49, 95% CI 0.16; 1.46, p = 0.129) and “wound dehiscence rate” (RR = 0.25, 95% CI 0.01; 4.27, p = 0.170) are detailed in the Supplementary Material, the forest plots are shown in Figures S4–S8, respectively.

## Risk-of-bias assessment

Risk of bias assessment was performed with the Rob-2 tool for RCTs and the ROBINS-I tool for the case–control study. Detailed results and domain and study level figures can be found in Supplementary Material, Figures S9–S17. Most of the studies presented some concerns due to lacking preregistered protocols. We found high-risk domains in two studies: the “randomization process” and "selection of the reported result" domains were considered a high bias risk in these trials.

## Publication bias and heterogeneity

Funnel plots can be found in the Supplementary Material, Figures S18–S25. Strong publication bias was not suspected in the examined outcomes.

Heterogeneity was mainly moderate in most of the analyses, but high heterogeneity was observed regarding seroma formation rates, the total volume of drainage, days to drain removal, and seroma aspiration rates. This is due to the difference in strategies used by the researchers, such as the intervention timing or choice for GC, etc.

## Certainty of evidence

For each comparison, the level of certainty of evidence ranged from very low to moderate. Supplementary Tables S3, 4 contain the results of the GRADE assessment. There was very low certainty regarding the “seroma formation rate” outcome due to a serious risk of bias and inconsistency. The low certainty of “wound infection rate” was because of the serious risk of bias and serious imprecision.

In most of the studies, due to high heterogeneity, the inconsistency varied from serious to very serious. We found no serious indirectness across the studies. Imprecision ranged from not serious to extremely serious due to the low number of analyzed studies on specific outcomes.

## Discussion

We aimed to investigate whether perioperative GCs are effective in preventing seroma formation after mastectomies. Based on the data from randomized controlled trials, our meta-analysis demonstrated that perioperative GC usage may lower seroma formation rates, decrease the total volume of drainage, and lead to earlier drain removal after breast cancer surgeries. These results are comparable with other interventions aimed at reducing seroma formation rates, such as quilting sutures or tranexamic acid use. In a recent study by Morarasu et al. [54], using quilting sutures during mastectomies was highly effective in reducing seroma formation rates.

Since the etiology of seroma formation is likely multifactorial, the prevention method must act in multiple pathways. Quilting sutures are effective due to the obliteration of dead space [55], but they do not affect the other factors. Surgery-induced stress response is a complex neurohumoral and immune reaction, and GCs may suppress hyperinflammatory reactions by increasing the transcription of anti-inflammatory proteins and decreasing the transcription of inflammatory cytokines and chemokines [56]. Decreased swelling and edema seen in maxillofacial surgeries [57] due to steroid administration may also be translated to the breast surgery setting. Furthermore, GCs may also reduce lymphatic fluid buildup by inducing lymphatic dysfunction, as proposed by Zhong et al. [58] These aforementioned properties of GCs may explain the high efficacy they showed in this study in preventing seroma formation.

GC administration reported by Talha et al. [51] significantly decreased C-reactive protein (CRP) and interleukin 6 values 24 h postoperatively compared to the placebo group. Albatanony et al. [40] only examined CRP, which was also considerably lower in the intervention group in the same timeframe. These results further support the theory that GCs attenuate the inflammatory response related to surgery.

There was no statistical difference between the intervention and control groups regarding wound infections. However, a clear tendency for higher surgical site infection rates was observed in the GC groups. While GC administration is a well-known risk factor for surgical site infections [59], the association between this complication and non-chronic perioperative GC use is debated in the literature [60]. A recent, large trial of patients undergoing non-cardiac surgery and receiving preoperative dexamethasone found no difference in wound infection rates between the GC and placebo groups [61]. In the study by Srinivasa et al. [22] assessing patients undergoing major abdominal surgeries, wound infection rates were lower in the intervention groups. Since antibiotic prophylaxis is mandatory in major abdominal surgeries according to enhanced recovery after surgery guidelines [62], this could hint at a potential solution if the higher wound infections observed in this study were to be verified.

Antibiotic prophylaxis in breast surgery remains a controversial topic. A recent study[63] found no difference in surgical site infection rates comparing groups with and without antibiotic therapy. Most of our included studies did not report on the use of antibiotics, Shiraz et al. [49] administered preoperative, while Albatanony et al. [40] used postoperative antibiotic prophylaxis, although they did not state wound infection rates in their respective studies.

Regarding secondary outcomes, perioperative GC usage could potentially decrease the volume and duration of postoperative drainage. Comparing it to other intervention methods, quilting sutures [54] showed a higher, while tranexamic acid [64] use demonstrated a lower efficacy regarding these aspects. Furthermore, fewer seroma aspirations, reduced first post-operative day drainage volumes, and lower wound necrosis and dehiscence rates were observed in the GC groups, although they did not reach statistical significance. The reduced need for aspirations leads to fewer outpatient clinic appearances, thus lessening psychophysiological burdens on the patients. Fewer wound complications spare the patients from undergoing additional surgical procedures, such as excision, re-suture, or negative pressure wound therapy.

Despite subgroup analysis for pre- and postoperative administration of GCs not showing significant difference, there was a tendency for preoperative usage to be the more effective compared to postoperative use. This effect is likely due to the early modulation of the inflammatory cascade, which is initiated immediately upon tissue injury during surgery; by administering GCs preoperatively, their anti-inflammatory and anti-exudative effects are present at the time of surgical trauma, thereby minimizing the development of seroma from the outset [65]. In contrast, postoperative administration may occur too late to suppress the initial inflammatory response adequately [45]. Since no study has compared the aforementioned GC application methods directly, this debate warrants future research.

A nonsignificant trend of lower wound infection rates in the postoperative subgroup was observed, which could be explained by the timing of immunosuppressive effects. Preoperative administration may transiently impair local immune responses during the critical early phase of wound healing, potentially increasing susceptibility to infection [65]. In contrast, postoperative administration, particularly when given locally into the cavity, may limit systemic immunosuppression while still providing anti-inflammatory benefits. These findings underscore the importance of carefully considering the timing and method of GC administration, particularly in patients at higher risk for postoperative infections. Further studies are warranted to confirm this trend and to optimize safety in clinical practice.

Despite not reaching statistical significance, HC showed the highest efficacy across the presented outcomes, except for days to drain removal. Studies using HC had the highest heterogeneity concerning drug administration, though: Talha et al. [51] doubled the dose of the steroids, while Albatanony et al. [40] used tranexamic acid in the intervention group as well. In a recent meta-analysis [64] assessing patients undergoing breast surgery, tranexamic acid use significantly reduced both seroma formation and total drainage volumes.

In our meta-analysis, many studies did not specify the axillary surgery type. Qvamme et al. [46] presented data on both AD and SLND patients: intracavitary MP treatment was beneficial in the SLND group but was not effective in preventing seroma formation in the AD group.

## Strengths and limitations

Regarding the strengths of our analysis, this study is the first comprehensive review on this topic. We successfully analyzed multiple outcomes and different subgroups. Except for the outcome “seroma aspiration”, we have conducted our meta-analysis of RCTs only, presenting a high level of evidence. We analyzed influence plots as well to identify any potential outliers. The risk of bias and publication bias were critically assessed.

Considering this work's limitations, different timings of the intervention, different drug choices or even a combination of drugs were used in some cases. We have attempted to tackle these limitations via subgroup analysis. Furthermore, the administration of additional drugs (such as antibiotics prophylaxis or preoperative dexamethasone) varied or was not reported accurately. The certainty of evidence ranged from low to moderate due to study limitations, inconsistency, and imprecision, which reduces confidence in the pooled estimates and limits strong conclusions. Serious to very serious inconsistency across studies, likely caused by differences in interventions, populations, and methods, further diminishes robustness and generalizability. Therefore, these results should be interpreted cautiously and underscore the need for more high-quality randomized, homogeneous studies to confirm these findings.

## Implications for practice and research

Clinicians should stratify patients preoperatively for seroma formation risk based on the aforementioned factors (e.g., age, BMI, and extent of axillary dissection). In high-risk patients meeting multiple risk criteria, perioperative GC administration may be considered. [66, 67]

Additional studies should verify the safety of perioperative GCs. If wound infection was a significant drawback of the treatment, antibiotic administration could prevent this complication. The possible synergistic effects of different interventions should also be explored, e.g., the combination of GCs and quilting sutures or tranexamic acid. The cost-effectiveness of GC application should also be explored in future studies. Further prospective data collection and multi-center randomized clinical trials are needed to assess the best drug choice, dose, and intervention timing.

## Conclusion

Based on our findings, perioperative glucocorticoid administration may reduce seroma formation rates, total drainage volume, and time to drain removal in patients undergoing mastectomy. However, there is also a possible increased risk of wound infections, which warrants further investigation.

## Supplementary Information

Below is the link to the electronic supplementary material.

Supplementary file1 (DOCX 5043 KB)

## Data Availability

The datasets used in this study can be found in the full-text articles included in the systematic review and meta-analysis.

## Abbreviations

AD: Axillary lymph node dissection
BMI: Body mass index
CI: Confidence interval
CRP: C-reactive protein
Figure S: Figure in Supplementary material
GC: Glucocorticoid
GRADE: Grades of Recommendation, Assessment, Development, and Evaluation
HC: Hydrocortisone
MD: Mean difference
MP: Methylprednisolone
MRM: Modified radical mastectomy
OR: Odds ratio
RCT: Randomized controlled trial
Rob 2: Risk of bias tool for randomized trials
RR: Risk Ratio
SD: Standard deviation
SLND: Sentinel lymph node dissection
