# Supplementary material for: The significance of perioperative glucocorticoids in the prevention of seroma formation after mastectomies: a systematic review and meta-analysis
Source: Breast Cancer Res Treat. 2025 Nov 25;215(1):13. doi: 10.1007/s10549-025-07830-3 (PMC12647319; doi:10.1007/s10549-025-07830-3)
Supplement: Supplementary file 1 — Supplementary file1 (DOCX 5043 KB) [file 10549_2025_7830_MOESM1_ESM.docx]

**SUPPLEMENTARY MATERIAL**

**TITLE**

The significance of perioperative glucocorticoids in the prevention of seroma formation after mastectomies: A systematic review and meta-analysis

**AUTHORS**

Dr. Levente Doleviczényi MD^1,2^, Dr. Lőrinc Frivaldszky MD^1,3,5^, Dr. Anett Rancz MD^1^, Dóra Léna Fedorcsák^1^, Boglárka Lilla Szentes^1^, Dr. Péter Hegyi MD, PhD, DSc^1,4,6^, Dr. Zoltán Klárik MD, PhD^1,2^

**AFFILIATIONS**

1. Centre for Translational Medicine, Semmelweis University, Budapest, Hungary
2. Department of Surgery, Transplantation and Gastroenterology, Semmelweis University, Budapest, Hungary
3. Department of Obstetrics and Gynecology, Semmelweis University, Budapest, Hungary
4. Institute of Pancreatic Diseases, Semmelweis University, Budapest, Hungary
5. MRE Bethesda Children's Hospital, Budapest, Hungary
6. Institute for Translational Medicine, Medical School, University of Pécs, Pécs, Hungary

**CORRESPONDING AUTHOR**

Dr. Zoltán Klárik MD, PhD

Postal address: Hungary 1082 Budapest, Üllői út 78.

Tel.: +36 20 825 1000

E-mail address: [klarik.zoltan@semmelweis.hu](mailto:klarik.zoltan@semmelweis.hu)

**CONTENT**

The supplementary material contains the PRISMA checklist, the detailed search key, further characteristics of the included studies, additional analyses for the outcomes “seroma aspiration rate”, “drainage on first postoperative day”, “wound necrosis rate” and “wound dehiscence rate”. Risk of bias analyses, certainty of evidence tables, funnel and influence plots can also be found in this material.

**Figure Legends**

**Figure S1.** Forest plot demonstrating seroma formation rates in the intervention and control groups. Subgroups based on the timing of intervention. N, number of patients in each arm; RR, risk ratio; CI, confidence interval.

**Figure S2.** Forest plot showing the total volume of drainage in the intervention and control groups. Subgroups based on the timing of intervention. N, number of patients in each arm; MD, mean difference; CI, confidence interval.

**Figure S3.** Forest plot demonstrating the length of drainage in the intervention and control groups. Subgroups based on the timing of intervention. N, number of patients in each arm; MD, mean difference; CI, confidence interval.

**Figure S4.** Forest plot demonstrating the effect of perioperative glucocorticoids on seroma aspiration rates in patients undergoing mastectomies. Subgroups based on the timing of intervention. OR, odds ratio; CI, confidence interval.

**Figure S5.** Forest plot demonstrating the effect of perioperative glucocorticoids on seroma aspiration rates in patients undergoing mastectomies. Subgroups based on glucocorticoid types. OR, odds ratio; CI, confidence interval.

**Figure S6.** Forest plot demonstrating the effect of perioperative glucocorticoids on 1^st^ post-operative day drainage volumes in patients undergoing mastectomies. N, number of patients in each arm; SD, standard deviation; MD, mean difference; CI, confidence interval.

**Figure S7.** Forest plot demonstrating the effect of perioperative glucocorticoids on wound necrosis rates in patients undergoing mastectomies. Subgroups based on the timing of intervention. N, number of patients in each arm; RR, risk ratio; CI, confidence interval.

**Figure S8.** Forest plot demonstrating the effect of perioperative glucocorticoids on wound dehiscence rates in patients undergoing mastectomies. Subgroups based on the timing of intervention. N, number of patients in each arm; RR, risk ratio; CI, confidence interval.

**Figure S9.** The risk of bias assessment at the study and domain level for the seroma formation rate.

**Figure S10.** The risk of bias assessment at the study and domain level for the wound infection rate.

**Figure S11.** The risk of bias assessment at the study and domain level for the total volume of drainage.

**Figure S12.** The risk of bias assessment at the study and domain level for the days to drain removal.

**Figure S13.** The risk of bias assessment at the study and domain level for the seroma aspiration rate (Rob-2 tool).

**Figure S14.** The risk of bias assessment at the study and domain level for the seroma aspiration rate (ROBINS-I tool).

**Figure S15.**The risk of bias assessment at the study and domain level for the 1^st^ post-operative day drainage volume.

**Figure S16.** The risk of bias assessment at the study and domain level for the wound necrosis rate.

**Figure S17.** The risk of bias assessment at the study and domain level for the wound dehiscence rate.

**Figure S18.** Funnel plot for seroma formation rate.

**Figure S19.** Funnel plot for wound infection rate.

**Figure S20.** Funnel plot for total volume of drainage.

**Figure S21.** Funnel plot for days to drain removal.

**Figure S22.** Funnel plot for seroma aspiration rate.

**Figure S23.** Funnel plot for drainage volume on 1^st^ postoperative day.

**Figure S24.** Funnel plot for wound necrosis rate.

**Figure S25.** Funnel plot for wound dehiscence rate.

**Figure S26.** Influence forest plot for seroma formation rate. CI, confidence interval.

**Figure S27.** Influence forest plot for wound infection rate. CI, confidence interval.

**Figure S28.** Influence forest plot for the total volume of drainage. CI, confidence interval.

**Figure S29.** Influence forest plot for days to drain removal. CI, confidence interval.

**Figure S30.** Influence forest plot for seroma aspiration rate. CI, confidence interval.

**Figure S31.** Influence forest plot for drainage volume on 1^st^ postoperative day. CI, confidence interval.

**Figure S32.** Influence forest plot for wound necrosis rate. CI, confidence interval.

**Figure S33.** Influence forest plot for wound dehiscence rate. CI, confidence interval.

**Table Legends**

**Table S1.** PRISMA checklist.

**Table S2.** The detailed search key.

**Table S3.** Further characteristics of the included studies (Age, BMI, study period, operation details).

**Table S4.** Detailed inclusion and exclusion criteria for the included studies.

**Table S5.** Summary of findings table of the quality of evidence for seroma formation and wound infection rates the total volume of drainage and day to drain removal.

**Table S6.** Summary of findings table of the quality of evidence for seroma aspiration rate, 1^st^ post-operative day drainage volume, wound necrosis, and dehiscence rates.

**Table S1. PRISMA checklist**

| **Section and Topic** | **Item #** | **Checklist item** | **Location where item is reported** |
| --- | --- | --- | --- |
| **TITLE** | | |  |
| Title | 1 | Identify the report as a systematic review. | 1 |
| **ABSTRACT** | | |  |
| Abstract | 2 | See the PRISMA 2020 for Abstracts checklist. | 2 |
| **INTRODUCTION** | | |  |
| Rationale | 3 | Describe the rationale for the review in the context of existing knowledge. | 3 |
| Objectives | 4 | Provide an explicit statement of the objective(s) or question(s) the review addresses. | 3 |
| **METHODS** | | |  |
| Eligibility criteria | 5 | Specify the inclusion and exclusion criteria for the review and how studies were grouped for the syntheses. | 4 |
| Information sources | 6 | Specify all databases, registers, websites, organisations, reference lists and other sources searched or consulted to identify studies. Specify the date when each source was last searched or consulted. | 4 |
| Search strategy | 7 | Present the full search strategies for all databases, registers and websites, including any filters and limits used. | 4 and Supplementary Table S2 |
| Selection process | 8 | Specify the methods used to decide whether a study met the inclusion criteria of the review, including how many reviewers screened each record and each report retrieved, whether they worked independently, and if applicable, details of automation tools used in the process. | 4 |
| Data collection process | 9 | Specify the methods used to collect data from reports, including how many reviewers collected data from each report, whether they worked independently, any processes for obtaining or confirming data from study investigators, and if applicable, details of automation tools used in the process. | 4 |
| Data items | 10a | List and define all outcomes for which data were sought. Specify whether all results that were compatible with each outcome domain in each study were sought (e.g. for all measures, time points, analyses), and if not, the methods used to decide which results to collect. | 4 |
|  | 10b | List and define all other variables for which data were sought (e.g. participant and intervention characteristics, funding sources). Describe any assumptions made about any missing or unclear information. | 4, Table 1, Supplementary Table S3 and Table S4 |
| Study risk of bias assessment | 11 | Specify the methods used to assess risk of bias in the included studies, including details of the tool(s) used, how many reviewers assessed each study and whether they worked independently, and if applicable, details of automation tools used in the process. | 4 |
| Effect measures | 12 | Specify for each outcome the effect measure(s) (e.g. risk ratio, mean difference) used in the synthesis or presentation of results. | 5 |
| Synthesis methods | 13a | Describe the processes used to decide which studies were eligible for each synthesis (e.g. tabulating the study intervention characteristics and comparing against the planned groups for each synthesis (item #5)). | 5 |
|  | 13b | Describe any methods required to prepare the data for presentation or synthesis, such as handling of missing summary statistics, or data conversions. | 5 |
|  | 13c | Describe any methods used to tabulate or visually display results of individual studies and syntheses. | 5 |
|  | 13d | Describe any methods used to synthesize results and provide a rationale for the choice(s). If meta-analysis was performed, describe the model(s), method(s) to identify the presence and extent of statistical heterogeneity, and software package(s) used. | 5 |
|  | 13e | Describe any methods used to explore possible causes of heterogeneity among study results (e.g. subgroup analysis, meta-regression). | 5 |
|  | 13f | Describe any sensitivity analyses conducted to assess robustness of the synthesized results. | 5 |
| Reporting bias assessment | 14 | Describe any methods used to assess risk of bias due to missing results in a synthesis (arising from reporting biases). | 7 |
| Certainty assessment | 15 | Describe any methods used to assess certainty (or confidence) in the body of evidence for an outcome. | 8 |
| **RESULTS** | | |  |
| Study selection | 16a | Describe the results of the search and selection process, from the number of records identified in the search to the number of studies included in the review, ideally using a flow diagram. | 6 (Figure 1) |
|  | 16b | Cite studies that might appear to meet the inclusion criteria, but which were excluded, and explain why they were excluded. | - |
| Study characteristics | 17 | Cite each included study and present its characteristics. | 6 and Table 1 |
| Risk of bias in studies | 18 | Present assessments of risk of bias for each included study. | Supplementary Figures S9-S17 |
| Results of individual studies | 19 | For all outcomes, present, for each study: (a) summary statistics for each group (where appropriate) and (b) an effect estimate and its precision (e.g. confidence/credible interval), ideally using structured tables or plots. | Figures 2-5 and supplementary Figures S1-S8 |
| Results of syntheses | 20a | For each synthesis, briefly summarise the characteristics and risk of bias among contributing studies. | Table 1 and Supplementary Figures S9-S17 |
|  | 20b | Present results of all statistical syntheses conducted. If meta-analysis was done, present for each the summary estimate and its precision (e.g. confidence/credible interval) and measures of statistical heterogeneity. If comparing groups, describe the direction of the effect. | Figures 2-5 and supplementary Figures S1-S8 |
|  | 20c | Present results of all investigations of possible causes of heterogeneity among study results. | - |
|  | 20d | Present results of all sensitivity analyses conducted to assess the robustness of the synthesized results. | - |
| Reporting biases | 21 | Present assessments of risk of bias due to missing results (arising from reporting biases) for each synthesis assessed. | - |
| Certainty of evidence | 22 | Present assessments of certainty (or confidence) in the body of evidence for each outcome assessed. | Supplementary Table S5-S6 |
| **DISCUSSION** | | |  |
| Discussion | 23a | Provide a general interpretation of the results in the context of other evidence. | 12-13 |
|  | 23b | Discuss any limitations of the evidence included in the review. | 13 |
|  | 23c | Discuss any limitations of the review processes used. | 13 |
|  | 23d | Discuss implications of the results for practice, policy, and future research. | 13 |
| **OTHER INFORMATION** | | |  |
| Registration and protocol | 24a | Provide registration information for the review, including register name and registration number, or state that the review was not registered. | 2 |
|  | 24b | Indicate where the review protocol can be accessed, or state that a protocol was not prepared. | 4 |
|  | 24c | Describe and explain any amendments to information provided at registration or in the protocol. | 4 |
| Support | 25 | Describe sources of financial or non-financial support for the review, and the role of the funders or sponsors in the review. | 17 |
| Competing interests | 26 | Declare any competing interests of review authors. | 17 |
| Availability of data, code and other materials | 27 | Report which of the following are publicly available and where they can be found: template data collection forms; data extracted from included studies; data used for all analyses; analytic code; any other materials used in the review. | 17 |

| **Table S2. The detailed search key** | |
| --- | --- |
| **Database** | **Search Key** |
| **Embase**  **(2948 hits)** | (mastectom* OR mammectom*) AND (steroid* OR glucocortico* OR ’methylprednisolone’ OR ’prednisolone’ OR ’medrol’ OR ’solu-medrol’ OR ’solumedrol’ OR ’depo-medrol’ OR ’depomedrol’ OR ’triamcinolone’ OR ’kenalog’ OR ’aristopan’ OR ’trivaris’ OR ’hydrocortisone’ OR ’cortef’ OR ’solu-cortef’ OR ’solucortef’ OR ’dexamethasone’ OR ’dexasone’ OR ’solurex’ OR ’baycadron’ OR ’cortisone’ OR ’prednisone’ OR ’betamethasone’ OR ’beclomethasone’ OR ’vamorolone’ OR ’deflazacort’ OR ’budesonide’ OR ’cortisone’ OR ’isoflupredone’ OR ’fluticasone’) |
| **CENTRAL**  **(311 hits)** | (mastectom* OR mammectom*) AND (steroid* OR glucocortico* OR "methylprednisolone" OR "prednisolone" OR "medrol" OR "solu-medrol" OR "solumedrol" OR "depo-medrol" OR "depomedrol" OR "triamcinolone" OR "kenalog" OR "aristopan" OR "trivaris" OR "hydrocortisone" OR "cortef" OR "solu-cortef" OR "solucortef" OR "dexamethasone" OR "dexasone" OR "solurex" OR "baycadron" OR "cortisone" OR "prednisone" OR "betamethasone" OR "beclomethasone" OR "vamorolone" OR "deflazacort" OR "budesonide" OR "cortisone" OR "isoflupredone" OR "fluticasone") |
| **PubMed**  **(775 hits)** | (mastectom* OR mammectom*) AND (steroid* OR glucocortico* OR "methylprednisolone" OR "prednisolone" OR "medrol" OR "solu-medrol" OR "solumedrol" OR "depo-medrol" OR "depomedrol" OR "triamcinolone" OR "kenalog" OR "aristopan" OR "trivaris" OR "hydrocortisone" OR "cortef" OR "solu-cortef" OR "solucortef" OR "dexamethasone" OR "dexasone" OR "solurex" OR "baycadron" OR "cortisone" OR "prednisone" OR "betamethasone" OR "beclomethasone" OR "vamorolone" OR "deflazacort" OR "budesonide" OR "cortisone" OR "isoflupredone" OR "fluticasone") |
| **Scopus**  **(2883 hits)** | (mastectom* OR mammectom*) AND (steroid* OR glucocortico* OR "methylprednisolone" OR "prednisolone" OR "medrol" OR "solu-medrol" OR "solumedrol" OR "depo-medrol" OR "depomedrol" OR "triamcinolone" OR "kenalog" OR "aristopan" OR "trivaris" OR "hydrocortisone" OR "cortef" OR "solu-cortef" OR "solucortef" OR "dexamethasone" OR "dexasone" OR "solurex" OR "baycadron" OR "cortisone" OR "prednisone" OR "betamethasone" OR "beclomethasone" OR "vamorolone" OR "deflazacort" OR "budesonide" OR "cortisone" OR "isoflupredone" OR "fluticasone") |
| **Web of Science**  **(406 hits)** | (mastectom* OR mammectom*) AND (steroid* OR glucocortico* OR "methylprednisolone" OR "prednisolone" OR "medrol" OR "solu-medrol" OR "solumedrol" OR "depo-medrol" OR "depomedrol" OR "triamcinolone" OR "kenalog" OR "aristopan" OR "trivaris" OR "hydrocortisone" OR "cortef" OR "solu-cortef" OR "solucortef" OR "dexamethasone" OR "dexasone" OR "solurex" OR "baycadron" OR "cortisone" OR "prednisone" OR "betamethasone" OR "beclomethasone" OR "vamorolone" OR "deflazacort" OR "budesonide" OR "cortisone" OR "isoflupredone" OR "fluticasone") |

**Notes:** During the systematic search, our search key had two domains: (I.) key terms for the population and (II.) expressions regarding GCs. "All text" setting was used while searching the CENTRAL database. Regarding the Web of Science, "Exact search" was used for reproducibility. We performed the search on Scopus using the "Title - abstract - keyword" setting.


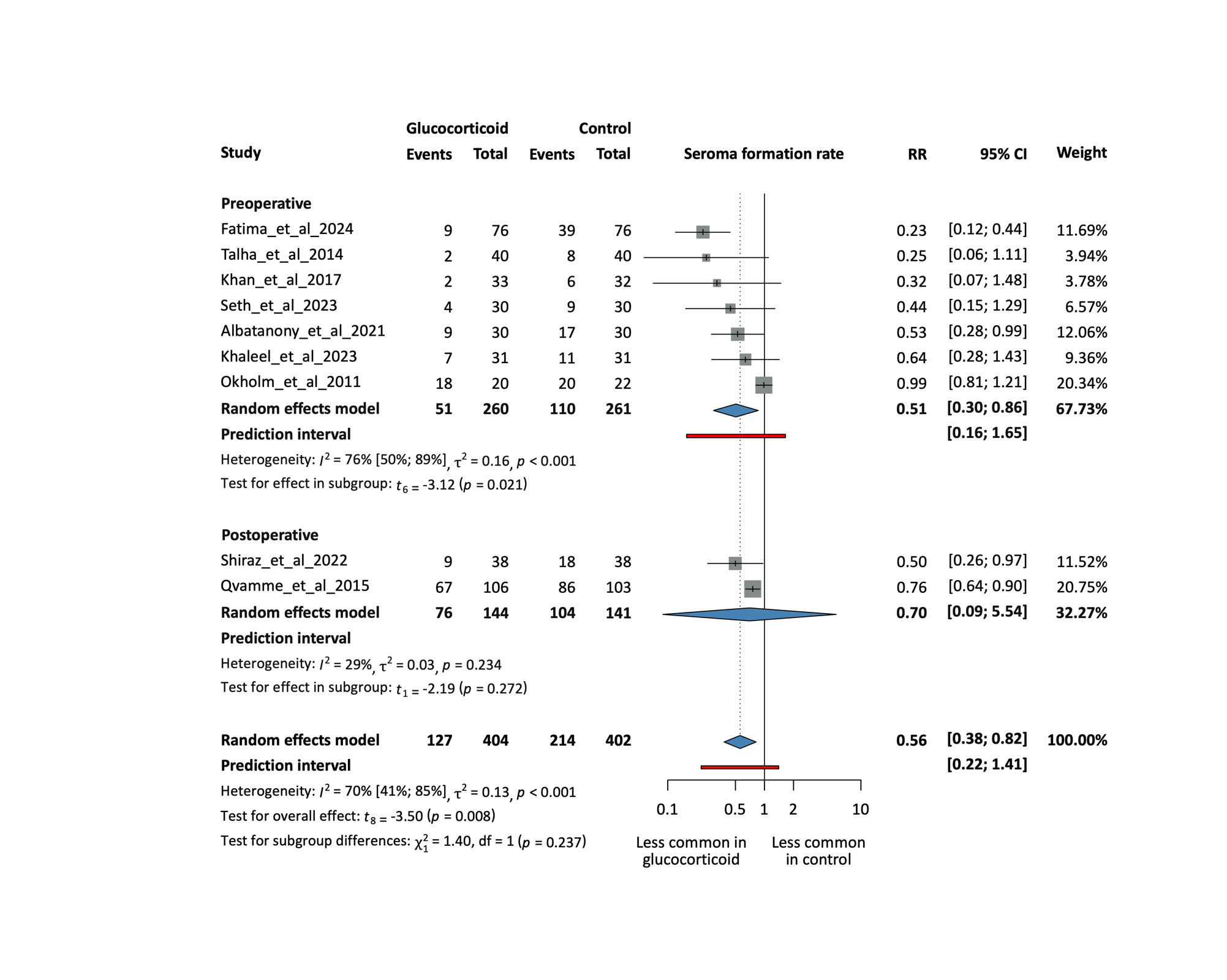


**Figure S1.** Forest plot demonstrating seroma formation rates in the intervention and control groups. Subgroups based on the timing of intervention. N, number of patients in each arm; RR, risk ratio; CI, confidence interval.


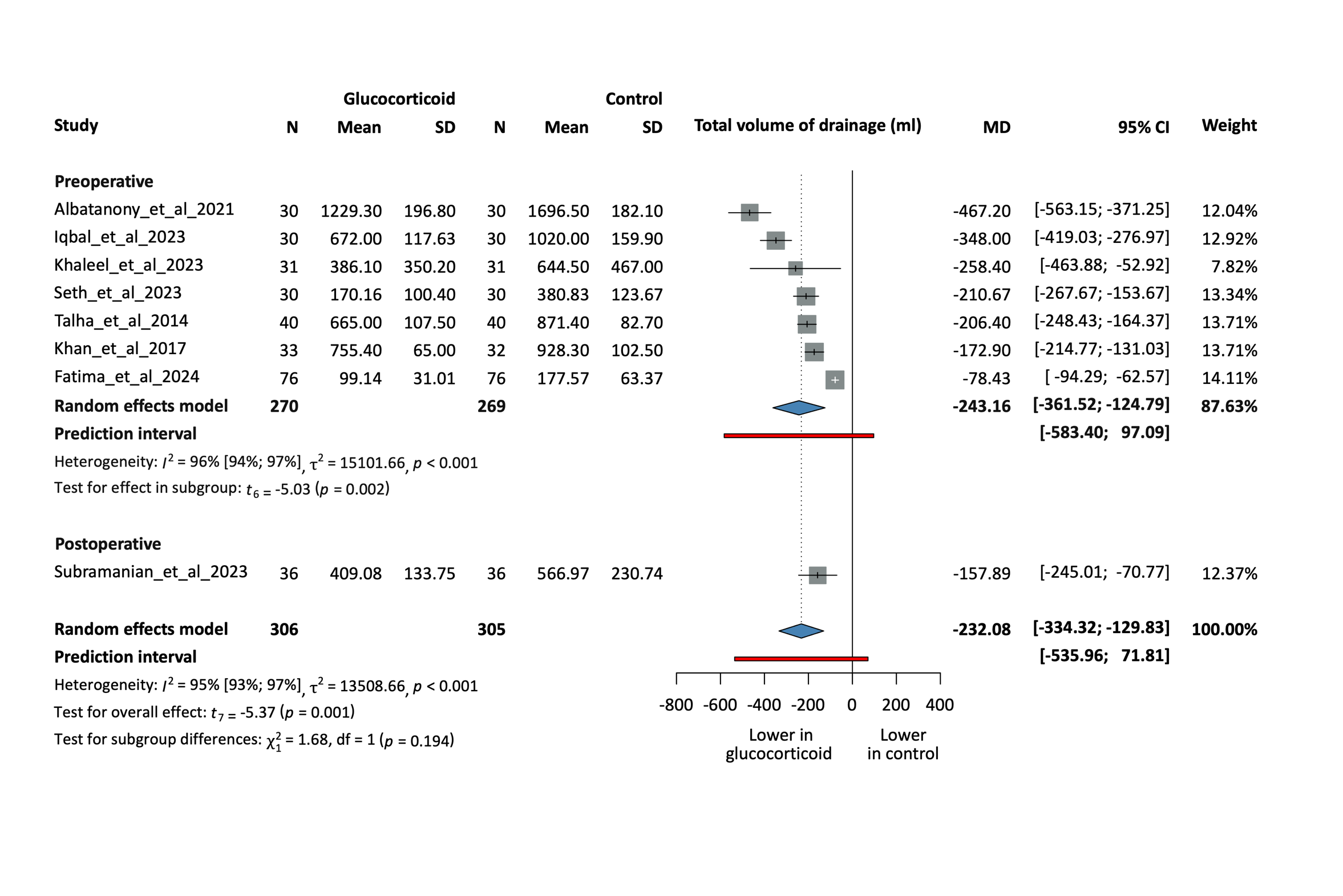


**Figure S2.** Forest plot showing the total volume of drainage in the intervention and control groups. Subgroups are based on the timing of intervention N, the number of patients in each arm, MD, mean difference, CI, and confidence interval.


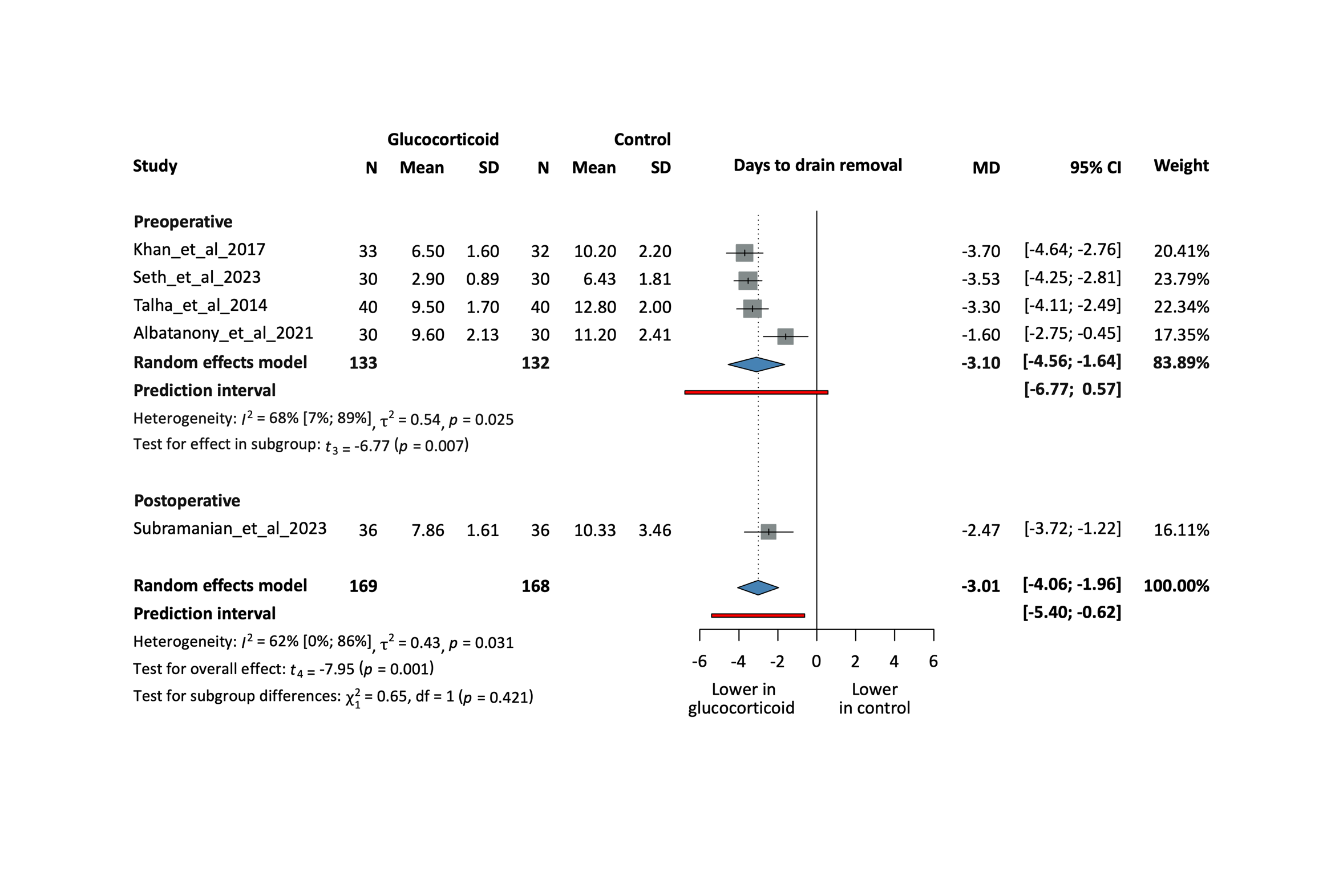


**Figure S3.** Forest plot demonstrating the length of drainage in the intervention and control groups. Subgroups based on the timing of intervention N, number of patients in each arm; MD, mean difference; CI, confidence interval.

**
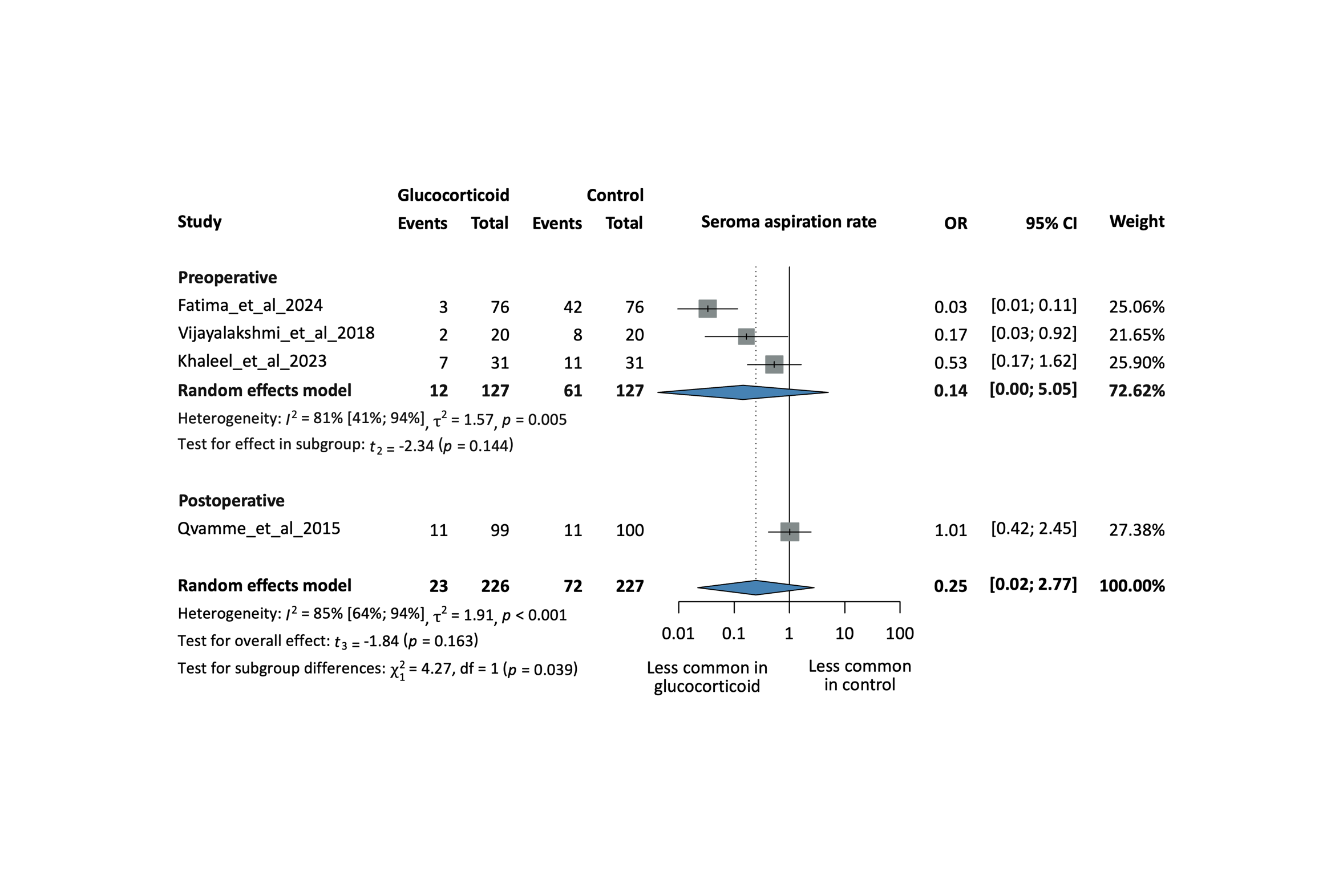
**

**Figure S4.** Forest plot demonstrating the effect of perioperative glucocorticoids on seroma aspiration rates in patients undergoing mastectomies. Subgroups based on the timing of intervention. OR, odds ratio; CI, confidence interval.

Three RCTs[1-3] and one case-control study[4] with 453 patients (226 in the intervention and 227 in the control groups) provided data on seroma aspirations. Our meta-analysis showed that seroma aspiration rates were lower in the glucocorticoid groups, although not reaching statistical significance (OR = 0.25, 95% CI: 0.02; 2.77, p = 0.163).

**
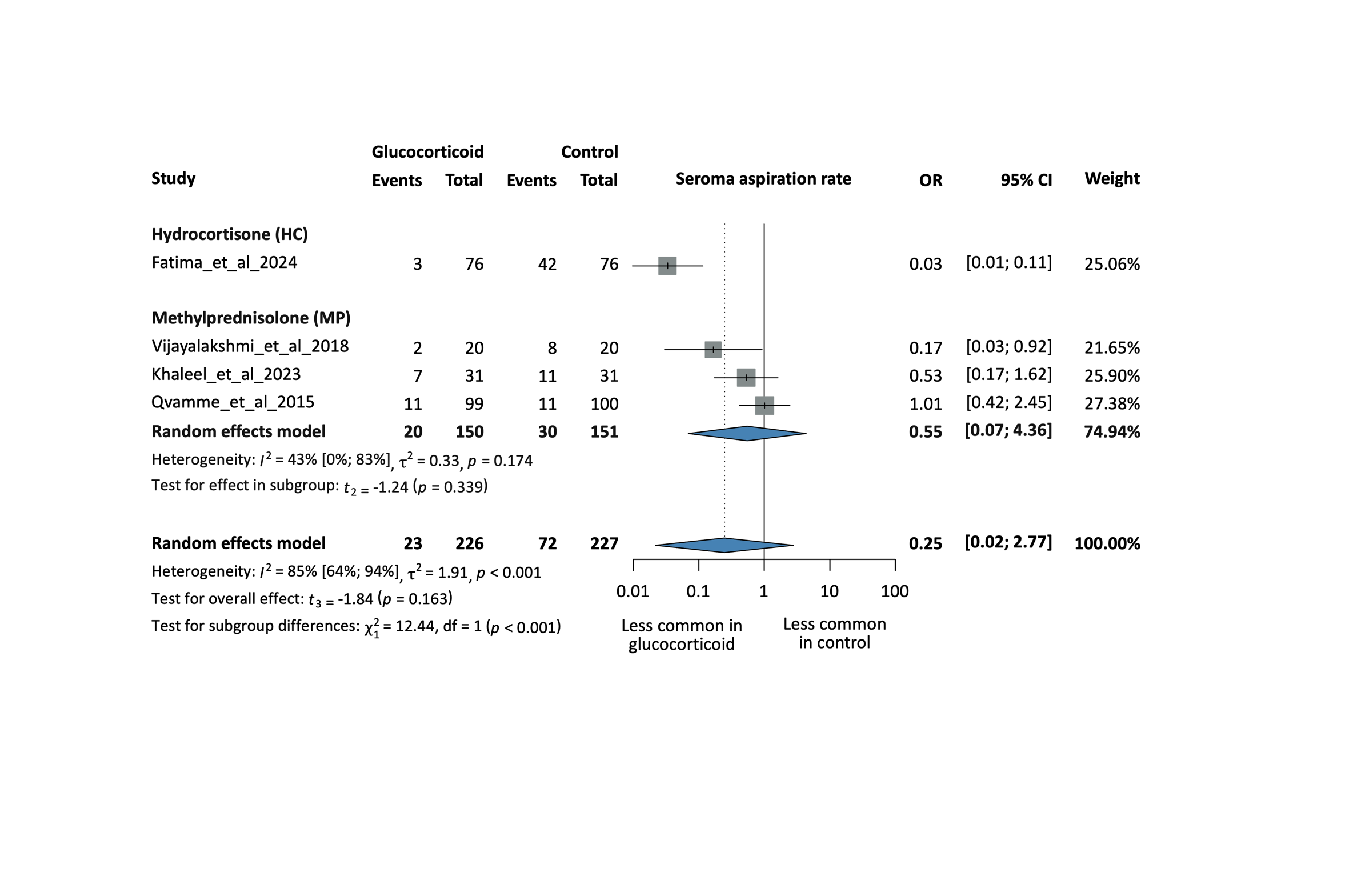
**

**Figure S5.** Forest plot demonstrating the effect of perioperative glucocorticoids on seroma aspiration rates in patients undergoing mastectomies. Subgroups based on glucocorticoid types. OR, odds ratio; CI, confidence interval.


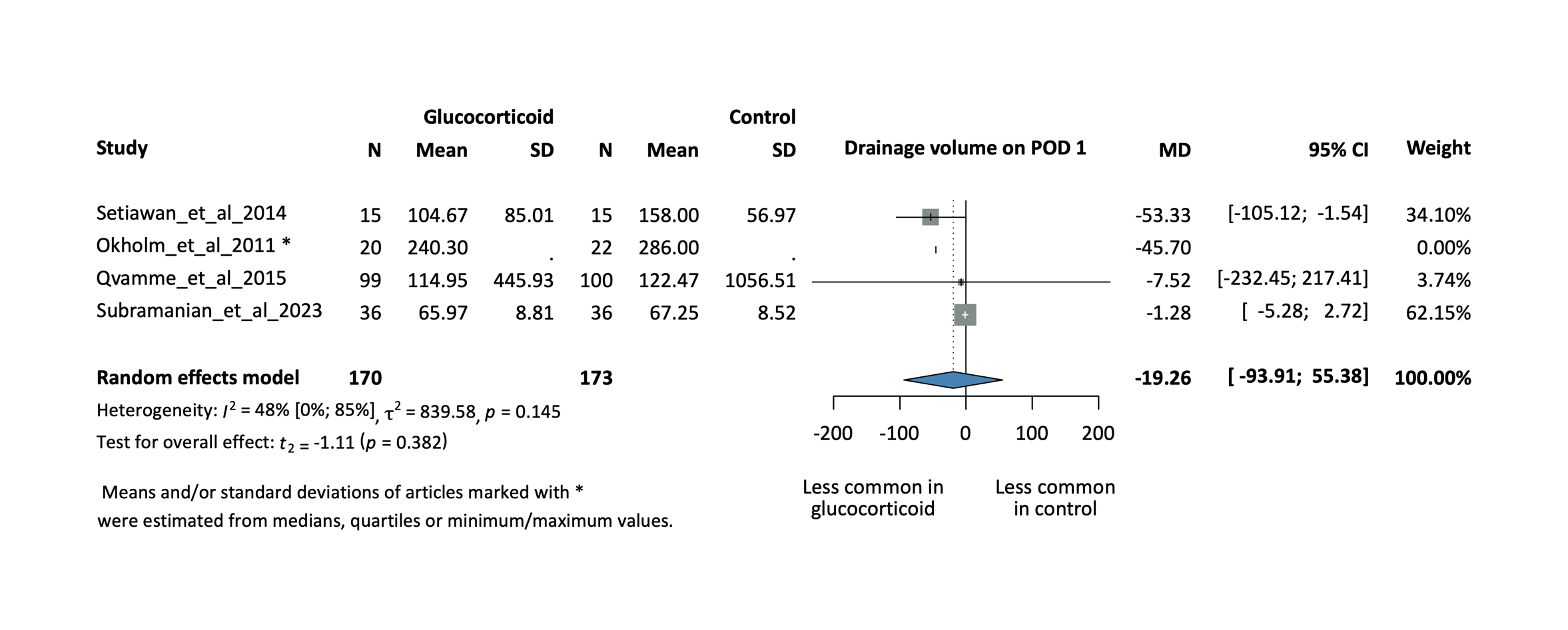


**Figure S6.** Forest plot demonstrating the effect of perioperative glucocorticoids on 1^st^ post-operative day drainage volumes in patients undergoing mastectomies. N, number of patients in each arm; SD, standard deviation; MD, mean difference; CI, confidence interval.

Analysis of four studies[3, 5-7], including 343 patients (170 in the intervention and 173 in the control groups), demonstrated lower 1^st^ post-operative day drainage volumes in the intervention groups (MD = -19.26 ml, 95% CI: -9391; -55.38, p = 0.382). The result was not statistically significant.

**
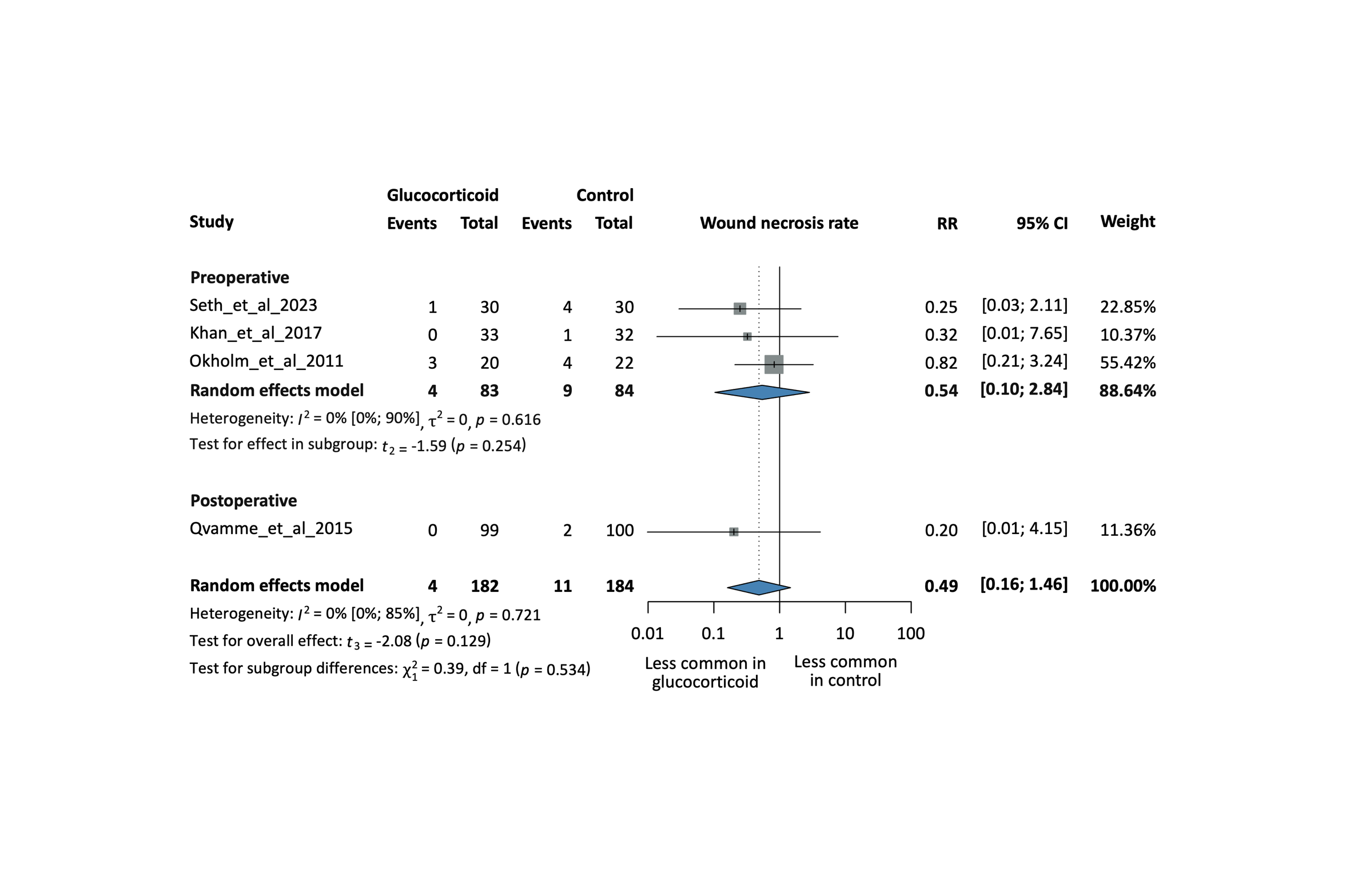
**

**Figure S7.** Forest plot demonstrating the effect of perioperative glucocorticoids on wound necrosis rates in patients undergoing mastectomies. Subgroups based on the timing of intervention. N, number of patients in each arm; RR, risk ratio; CI, confidence interval.

Four trials[3, 6, 8, 9] and 366 patients (182 in the glucocorticoid and 184 in the control group) were analyzed. Lower wound necrosis rates were observed in the intervention groups (RR = 0.49, 95% CI: 0.16; 1.46, p = 0.129); the result hasn't reached statistical significance.

**
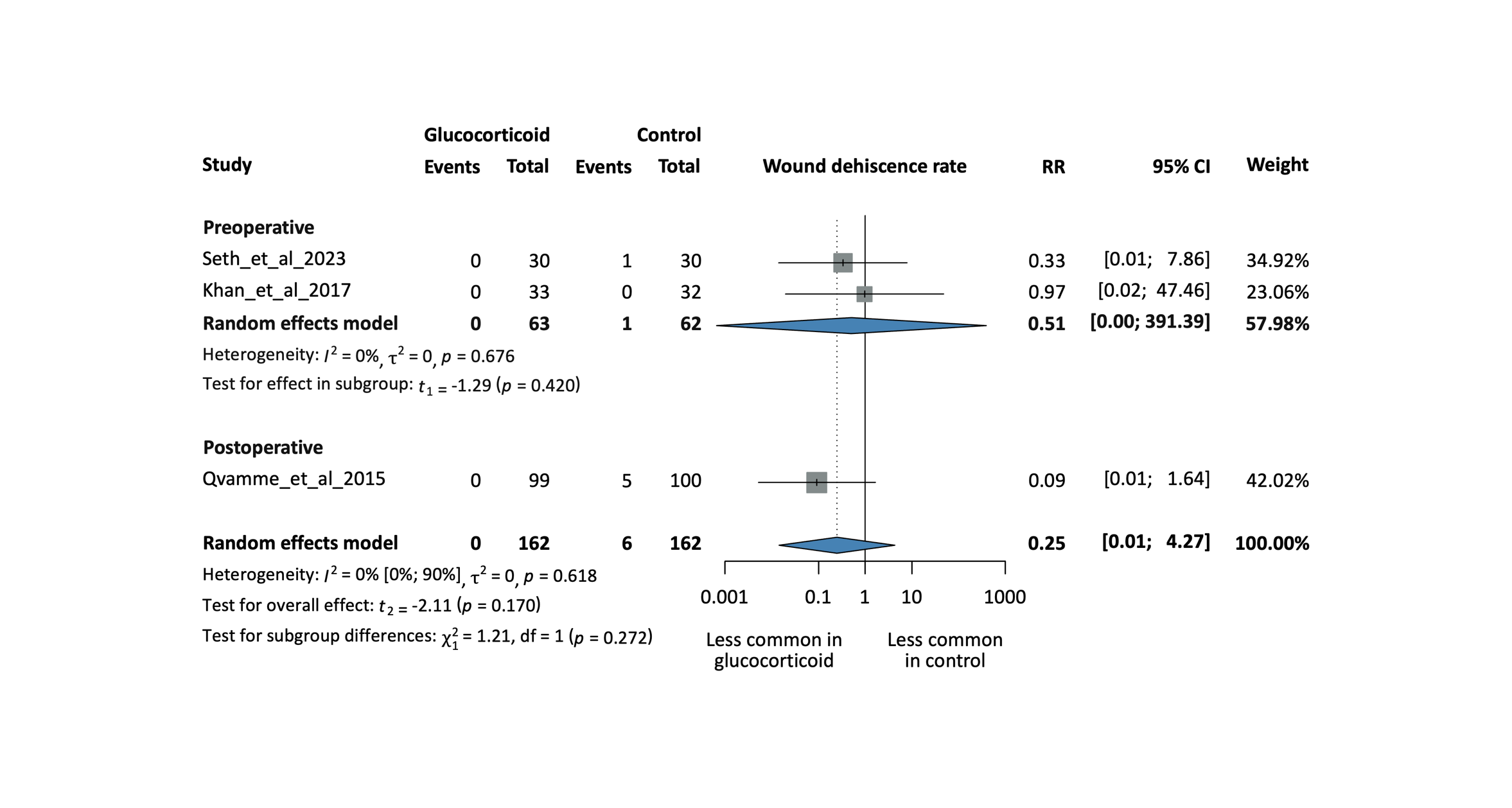
**

**Figure S8.** Forest plot demonstrating the effect of perioperative glucocorticoids on wound dehiscence rates in patients undergoing mastectomies. Subgroups based on the timing of intervention. N, number of patients in each arm; RR, risk ratio; CI, confidence interval.

Meta-analysis of three studies[3, 8, 9] with 324 patients (162 patients in both groups) showed lower wound dehiscence rates in the glucocorticoid groups, but the result was not significant statistically (RR = 0.25, 95% CI: 0.01; 4.27, p = 0.170).

**Table S3.** Further characteristics of the included studies (Age, BMI, study period, operation details).

| **Author (year)** | **Age**^†^ **(intervention)** | **Age**^†^ **(control)** | **BMI**^†^ **(intervention)** | **BMI**^†^ **(control)** | **Study period** | **Drains placed** | **Dissection method** | **Additional information** |
| --- | --- | --- | --- | --- | --- | --- | --- | --- |
| Albatanony et al. 2021[10] | ND | ND | ND | ND | 2020.01.-2020.05. | ND | ND | Postoperative antibiotics administration |
| Fatima et al. 2024[1] | 48,42±10,15 | 47,67±10,75 | ND | ND | 2021.01.-2021.12. | ND | Monopolar diathermy and sharp dissection | Compression dressing for 10 days and early arm physiotherapy |
| Iqbal et al. 2023[11] | ND | ND | ND | ND | ND | 2 | Steel scalpel | NA |
| Khaleel et al. 2023[2] | ND | ND | 23,6 | 24,1 | 2019.12.-2022.01. | ND | ND | NA |
| Khan et al. 2017[9] | 32,3±9,1 | 34,2±10,1 | ND | ND | 2012.01.-2014.04. | 1 | ND | NA |
| Okholm et al. 2011[6] | 63,2 (48-86) | 62,3 (43-79) | 23,9 (16-36) | 24 (20,4-31,1) | ND | 1 | Diathermy and sharp dissection | NA |
| Qvamme et al. 2015[3] | 63 (60-66) | 64 (61-67) | 24 (24–25) | 26 (24–27) | 2010.08.-2013.04. | 1 | Electrocautery and sharp dissection | Drain removed on 1^st^ postoperative day |
| Seth et al. 2023[8] | 40.1±8.1 | 42.1±8.2 | ND | ND | 2020.11.-2021.04. | 2 | ND | NA |
| Setiawan et al. 2014[5] | 50,27±9,65 | 46,27±6,76 | 24,15±1,88 | 24,05±1,74 | 2013.04.-2013.07. | ND | ND | NA |
| Shiraz et al. 2022[12] | 38±12,72 | 40±11,83 | ND | ND | 2019.10.-2020.04. | 1 | ND | Antibiotic prophylaxis, drain removed on 1^st^ postoperative day |
| Subramanian et al. 2023[7] | 53,75 | 52,47 | ND | ND | 2018.01.-2019.06. | 1 | ND | NA |
| Talha et al. 2014[13] | 49,1±10,2 | 48±9,1 | ND | ND | 2013.02.-2014.06. | 1 | Diathermy and sharp dissection | NA |
| Vijayalakshmi et al. 2018[4] | ND | ND | ND | ND | ND | 1 | ND | NA |

† parameters represented as mean with standard deviation, or median with range (minimum and maximum)

ND: not defined; NA: not applicable

Nine studies[1, 2, 4, 6, 8-11, 13] chose the preoperative methods. The administration of GC ranged from 1,5 hours prior to anesthesia to induction, although most trials did not specify the exact timing of the injection. Three studies[3, 7, 12] used the drugs in a postoperative setting. Shiraz et al.[12] and Qvamme et al.[3] removed the drains regardless of drainage volumes on the first postoperative day (POD) and injected methylprednisolone (MP) into the cavity. Subramanian et al.[7] clamped the drain for 8 hours after the injection on the 1^st^ POD. The trial by Setiawan et al. [5] did not specify the intervention timing.

Nine studies[2-9, 11, 12] used MP as the choice of drug. Intravenous doses ranged from 120 to 125 mgs, while 80 mgs of GC dissolved in 10 ml of NaCl were injected postoperatively. Three RCTs[1, 10, 13] administered hydrocortisone (HC). Fatima et al.[1] gave 100 mg of HC intravenously, while Talha et al.[13] repeated the dose preoperatively. Only these studies differed significantly in GC doses, so dose-response relationships could not be explored. Besides the GC mentioned above, Albatanony et al.[10] even used 5 ml of tranexamic acid.

Modified radical mastectomies (MRM) were performed dominantly; most of the studies did not differentiate between sentinel lymph node dissections (SLND) and axillary dissections (AD). Dissection was done mainly via diathermy and sharp dissection regarding the axilla, and one to two closed suction drains were placed in the included studies.

**Table S4.** Detailed inclusion and exclusion criterias of the included studies.

| **Author (year)** | **Inclusion criteria** | **Exclusion Criteria** |
| --- | --- | --- |
| Albatanony et al. 2021[10] | Female patient with operable cancer breast prepared for MRM was included in the study. | Patients with recurrent breast lesion, breast reconstruction, conservative breast surgery and patients receive neo-adjuvants therapy were excluded from the study. |
| Fatima et al. 2024[1] | All female patients aged 18 to 70 years, with primary breast cancer and axillary lymph node involvement were included and scheduled for MRM on an elective basis. | Patients were excluded from the study if they had stage IV breast cancer, had evidence of local infection, history of previous axillary surgery in the last six months, or had been treated with steroids within the last month before surgery, including inhalationalproducts. All those patients who were pregnant, allergic, or hypersensitive to hydrocortisone were omitted from the study. |
| Iqbal et al. 2023[11] | Inclusion criteria includes female Patients who were planned for MRM with age range of 30 to 60 years. | Patients with chronic medical comorbid conditions (diabetes mellitus, renal or hepatic insufficiency and coronary artery disease), CAB stage III or IV as per AJCC staging system, already on steroids, previous history of radiotherapy, chemotherapy or breast surgery and pregnant or lactating mothers were excluded from the study. |
| Khaleel et al. 2023[2] | All patients of Carcinoma Breast undergoing Modified Radical Mastectomy. Patients with carcinoma breast who received neoadjuvant chemotherapy followed by Modified Radical Mastectomy. | Patients with Diabetes mellitus, patients treated with glucocorticoids within 1 month before surgery, patients unwilling for study, carcinoma Breast Patients who didn’t undergo surgery, |
| Khan et al. 2017[9] | Patients who presented with symptoms of Breast Cancer were evaluated by taking history, examination; radiological workup and biopsy to confirm the diagnosis. Those patients who presented with 1st, 2nd and 3rd stage of breast cancer was included in the study. | Those with stage 4, recurrent disease, bleeding disorders or hypertensive were excluded from the study. |
| Okholm et al. 2011[6] | Women with operable primary breast cancer scheduled for mastectomy and axillary dissection. | Men, treatment with glucocorticoids within the past month, pregnancy, ischaemic heart diseases, diabetes, uraemia, treatment with carbamazepin, phenytoin, phenobarbital, rifam picin, salicylats and ciclosporin, history with psychoses. |
| Qvamme et al. 2015[3] | Eligible women were aged 18–70 years and scheduled for surgery (mastectomy and sentinel lymph node dissection or axillary dissection) for stage I–IIIA breast cancer at Copenhagen University Hospital Herlev or Rigshospitalet, Copenhagen, Denmark. Signed informed consent. | Previous axillary surgery (made within <4 months), recent (<1 month) treatment with systemic steroids, allergy to trial drug ingredients, pregnancy, inability to understand Danish, evidence of other relevant medical conditions judged to be inconsistent with participation. |
| Seth et al. 2023[8] | Comprised female patients aged 30-60 years having American Society of Anaesthesiologists (ASA) physical status classification system I or II, undergoing unilateral MRM with axillary lymph node dissection under general anaesthesia. All patients were tested for coronavirus disease-2019 (COVID-19) via nasal swab for polymerase chain reaction (PCR) test, and only those with negative results in the preceding 48 hours were included. | Pregnancy, known allergy to steroids, weighing <30kg, and patients with significant cardiovascular, pulmonary, neurological, hepatic, renal and metabolic disease, such as those with known diabetes and having uncontrolled glucose levels with a fasting blood glucose level >110mg/dl, known hypertensive with systolic blood pressure (SBP) of >140mmHg, those with ejection fraction (EF) of <40%, with ischaemic heart disease (IHD) and forced expiratory volume for 1 second (FEV1) <70 % of the normal in patients with pulmonary disease as per history and clinical record, were excluded. Patients with metastatic disease were also excluded. |
| Setiawan et al. 2014[5] | Female patients with a diagnosis of locally advanced breast cancer and mastectomy surgery. | Exclusion criteria were comorbid diseases of hypertension and diabetes mellitus. |
| Shiraz et al. 2022[12] | All women in age range 20-60 years diagnosed with carcinoma breast and booked in for modified radical mastectomy were included in the study. | Patients with a BMI>27, comorbidities including diabetes, hypertension, asthma and conditions requiring systemic steroid therapy were excluded from this study. |
| Subramanian et al. 2023[7] | Patients with carcinoma breast were included in the study after obtaining appropriate written consent and approval from the institutional ethics committee (Date of approval 21 December 2017). | Patients who underwent mastectomy without ALND, previous axillary surgery, pregnancy, and patients on anticoagulants or systemic steroids or immunocompromised were excluded from the study. |
| Talha et al. 2014[13] | The study included female patients with primary operable breast cancer who were admitted to the hospital of the Medical Research Institute, University of Alexandria, Egypt, during the period from February 2013 to June 2014 and scheduled for a modifi ed radical mastectomy. | Patients with endocrine or metabolic disorders, diabetes, morbid obesity (BMI >40 kg/m2 or 35 kg/m2 with comorbidity), history of central nervous system diseases, who were under current or recent medication affecting the sympathetic response or hormonal secretion, such as carbamazepin, phenytoin, phenobarbital, rifampicin, salicylats, and ciclosporin, patients with distant metastasis, and patients who received previous anticancer treatment were excluded from the study. |
| Vijayalakshmi et al. 2018[4] | Women with primary breast cancer, undergoing a mastectomy with either sentinel node biopsy or complete axillary dissection. | Men, treatment with glucocorticoids within the last month before surgery, including inhalation products, pregnancy, not able to speak Danish, severe heart disease, treatment with carbamazepine, phenytoin, phenobarbital, rifampicin, salicylates and ciclosporin, uremia, diabetes, other medical conditions, evaluated by the investigator, that make the patient unfit for participation, previous psychosis immunocompromised patients who have an indolent course. |

**
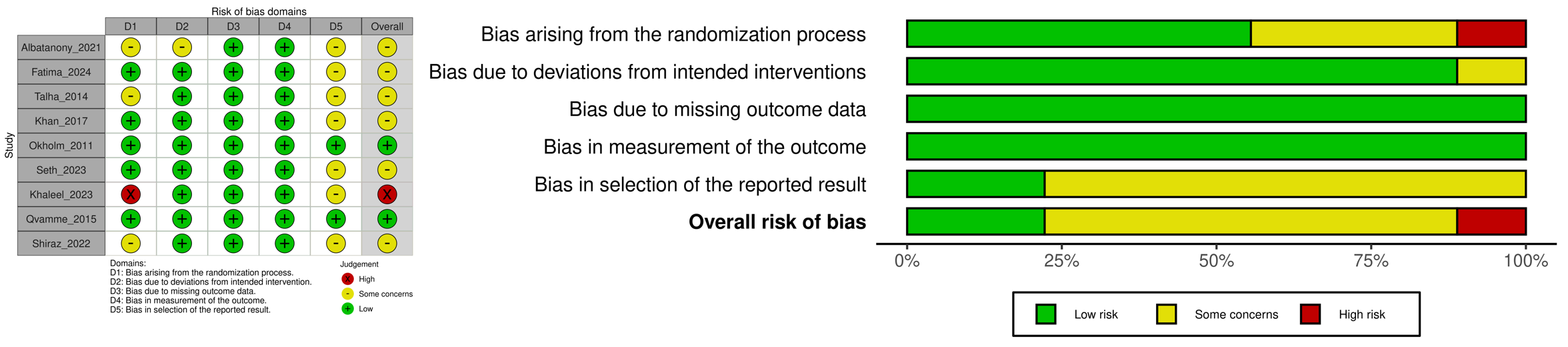
**

**Figure S9.** The risk of bias assessment at the study and domain level for the seroma formation rate.

**
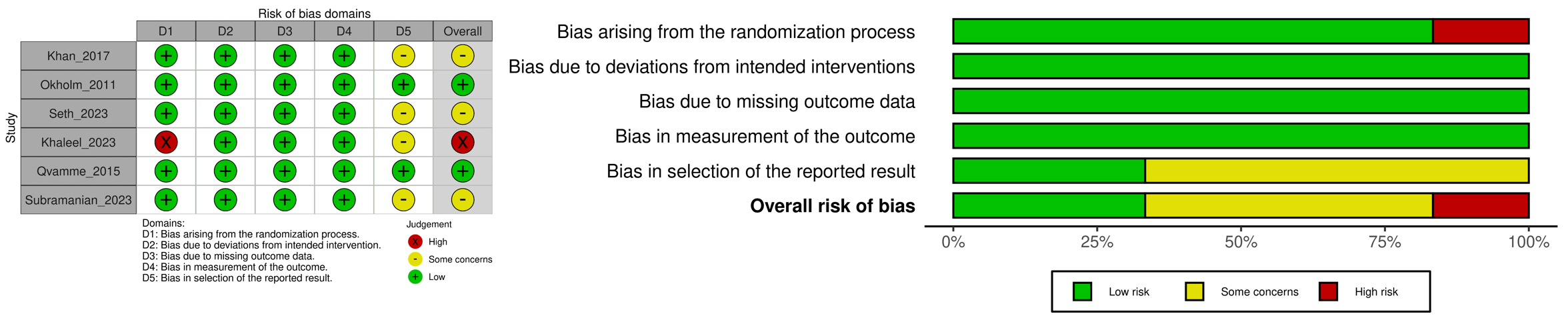
**

**Figure S10.** The risk of bias assessment at the study and domain level for the wound infection rate.

**
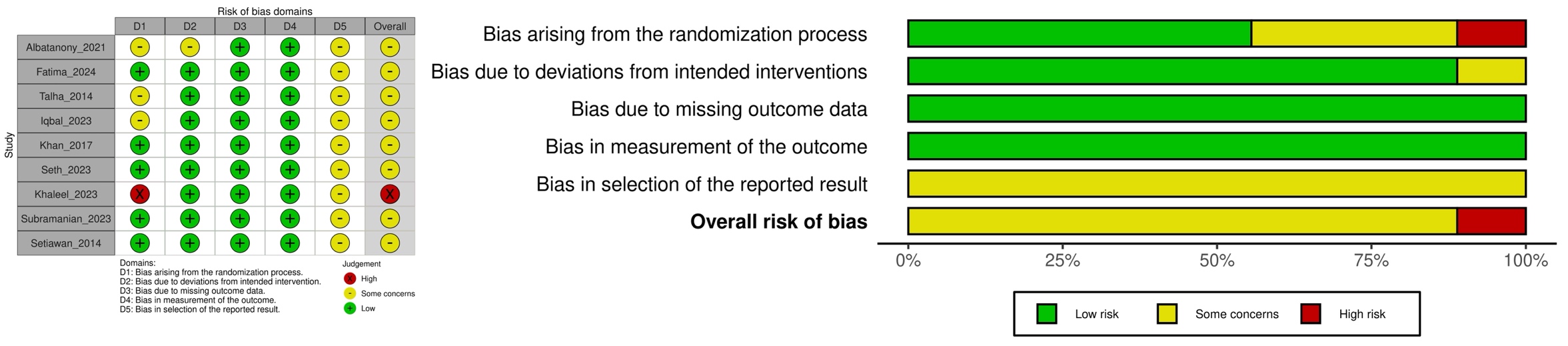
**

**Figure S11.** The risk of bias assessment at the study and domain level for the total volume of drainage.


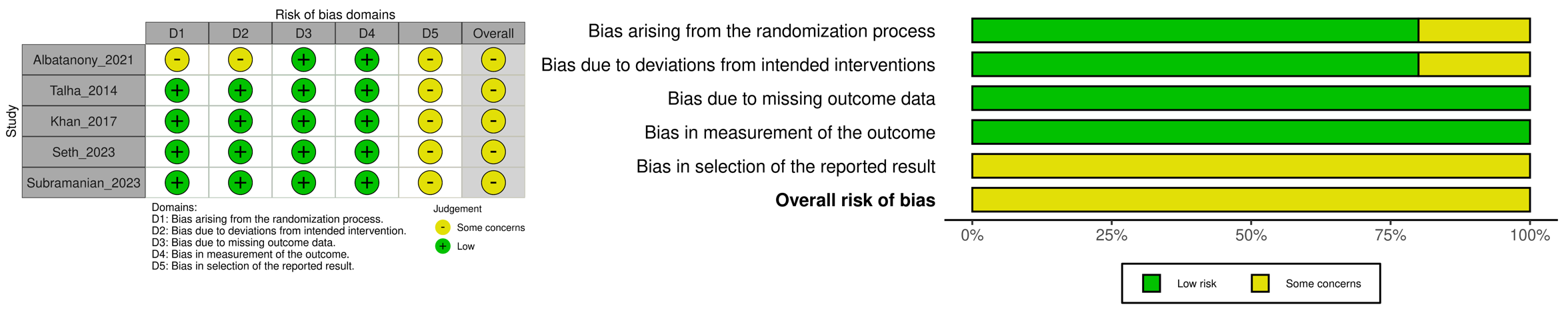


**Figure S12.** The risk of bias assessment at the study and domain level for the days to drain removal.

**
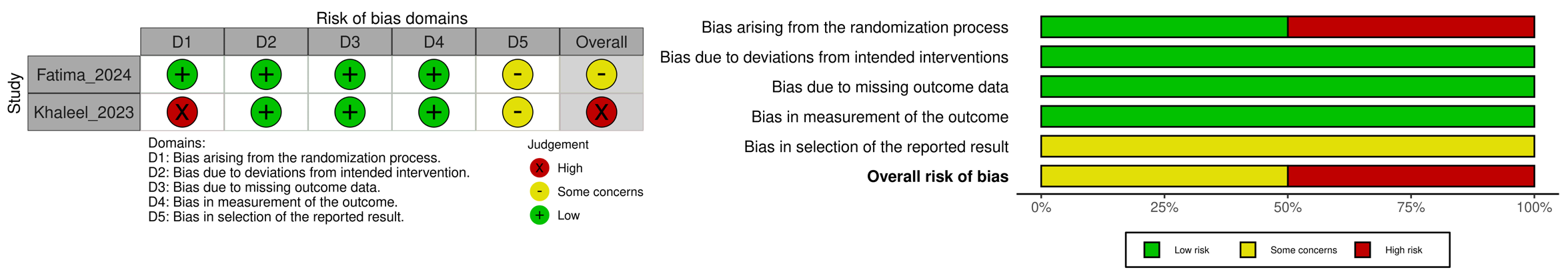
**

**Figure S13.** The risk of bias assessment at the study and domain level for the seroma aspiration rate (Rob-2 tool).

**
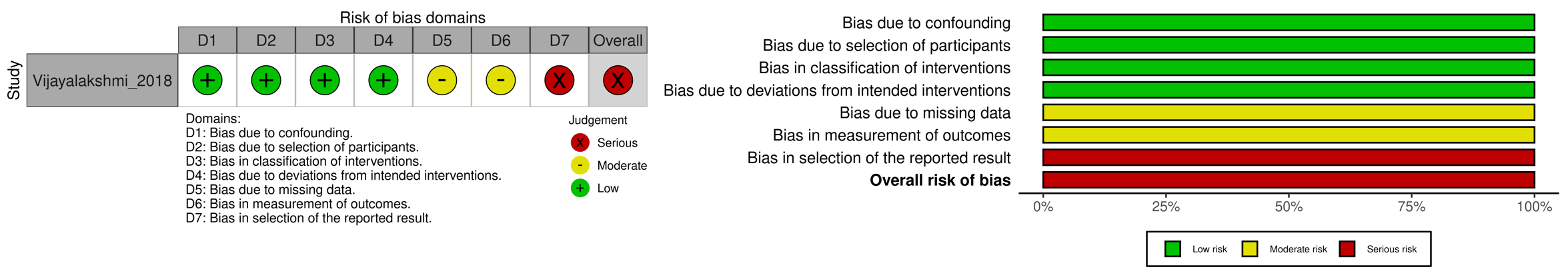
**

**Figure S14.** The risk of bias assessment at the study and domain level for the seroma aspiration rate (ROBINS-I tool).

**
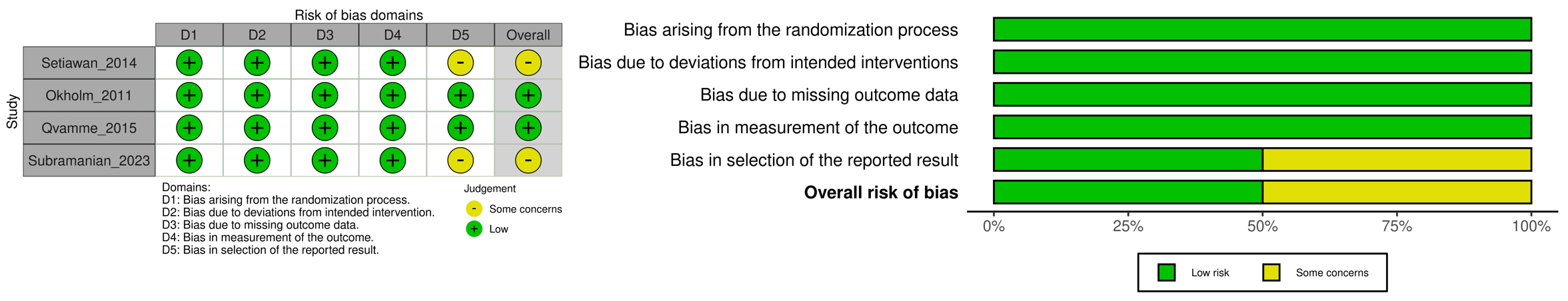
**

**Figure S15.**The risk of bias assessment at the study and domain level for the 1^st^ post-operative day drainage volume.


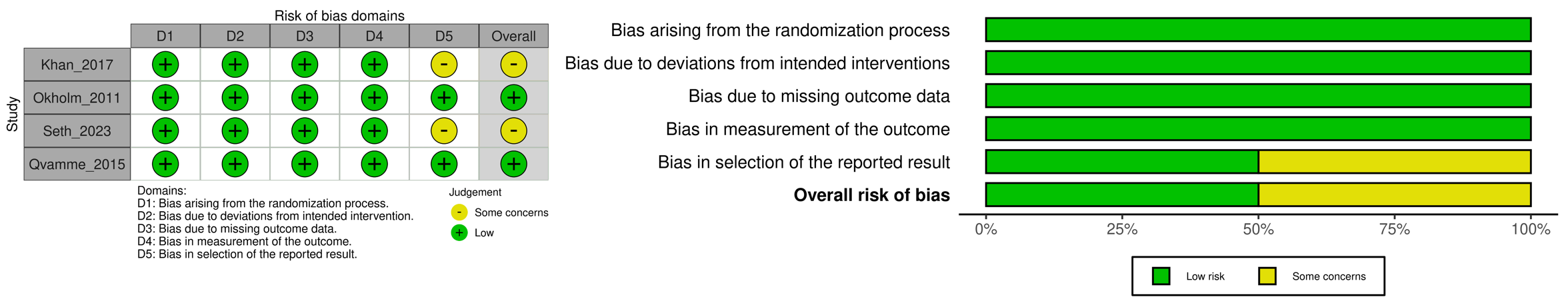


**Figure S16.** The risk of bias assessment at the study and domain level for the wound necrosis rate.

**
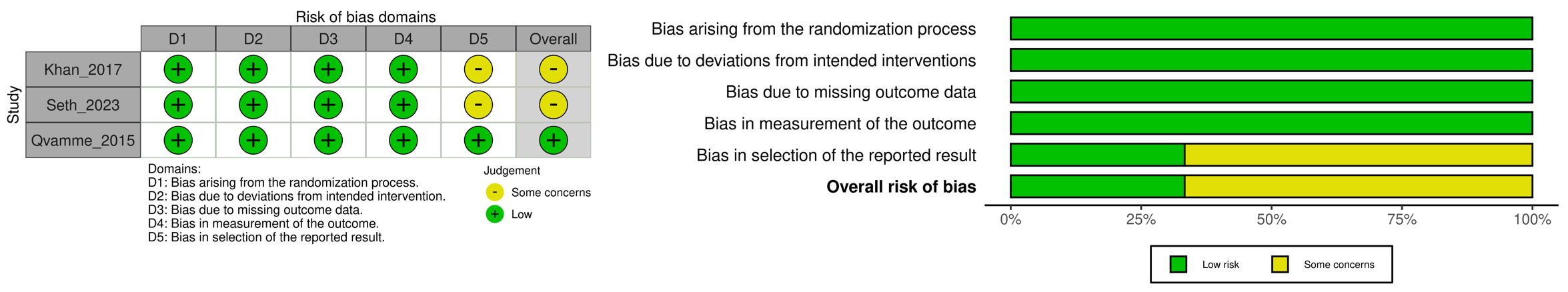
**

**Figure S17.** The risk of bias assessment at the study and domain level for the wound dehiscence rate.

**Table S5.** Summary of findings table of the quality of evidence for seroma formation and wound infection rates the total volume of drainage and day-to-drain removal.

| **Certainty assessment** | | | | | | | **№ of patients** | | **Effect** | | **Certainty** | **Importance** |
| --- | --- | --- | --- | --- | --- | --- | --- | --- | --- | --- | --- | --- |
| **№ of studies** | **Study design** | **Risk of bias** | **Inconsistency** | **Indirectness** | **Imprecision** | **Other considerations** | **glucocorticoids** | **control** | **Relative (95% CI)** | **Absolute (95% CI)** |  |  |
| **Seroma formation rate (assessed with: RR)** | | | | | | | | | | | | |
| 9 | randomised trials | serious^a^ | very serious^b^ | not serious | not serious | none | 127/404 (31.4%) | 214/402 (53.2%) | **RR 0.56** (0.38 to 0.82) | **234 fewer per 1,000** (from 330 fewer to 96 fewer) | ⨁◯◯◯ Very low^a,b^ | CRITICAL |
| **Wound infection rate (assessed with: RR)** | | | | | | | | | | | | |
| 6 | randomised trials | serious^a^ | not serious | not serious | serious^c^ | none | 18/249 (7.2%) | 14/251 (5.6%) | **RR 1.26** (0.82 to 1.92) | **15 more per 1,000** (from 10 fewer to 51 more) | ⨁⨁◯◯ Low^a,c^ | CRITICAL |
| **Total volume of drainage (assessed with: MD)** | | | | | | | | | | | | |
| 9 | randomised trials | serious^a^ | very serious^d^ | not serious | not serious | none | 321 | 320 | - | MD **213.36 ml lower** (321.5 lower to 114.22 lower) | ⨁◯◯◯ Very low^a,d^ | CRITICAL |
| **Days to drain removal (assessed with: MD)** | | | | | | | | | | | | |
| 5 | randomised trials | not serious | very serious^e^ | not serious | not serious | none | 169 | 168 | - | MD **3.01 days lower** (4.06 lower to 0.62 lower) | ⨁⨁◯◯ Low^e^ | CRITICAL |

**CI:** confidence interval; **MD:** mean difference; **RR:** risk ratio

#### Explanations

a. In most studies included, there were at least some concerns regarding the risk of bias. Therefore, the authors have decided to downgrade the level of evidence.

b. Heterogeneity with the I2 test was 70% (41%-85%), corresponding to a serious inconsistency. (0-25% low level of heterogeneity = not serious inconsistency, 25-50% moderate level of heterogeneity = serious inconsistency, 50% < high level of heterogeneity = very serious inconsistency).

c. Considering that the 95% CI fails to exclude important benefit or important harm, the authors decided to rate down for imprecision.

d. Heterogeneity with the I2 test was 95% (92%-97%), corresponding to a very serious inconsistency. (0-25% low level of heterogeneity = not serious inconsistency, 25-50% moderate level of heterogeneity = serious inconsistency, 50% < high level of heterogeneity = very serious inconsistency).

e. Heterogeneity with the I2 test was 62% (0%-86%), corresponding to a very serious inconsistency. (0-25% low level of heterogeneity = not serious inconsistency, 25-50% moderate level of heterogeneity = serious inconsistency, 50% < high level of heterogeneity = very serious inconsistency).

**Table S6.** Summary of findings table of the quality of evidence for seroma aspiration rate, 1^st^ post-operative day drainage volume, wound necrosis, and dehiscence rates.

| **Certainty assessment** | | | | | | | **№ of patients** | | **Effect** | | **Certainty** | **Importance** |
| --- | --- | --- | --- | --- | --- | --- | --- | --- | --- | --- | --- | --- |
| **№ of studies** | **Study design** | **Risk of bias** | **Inconsistency** | **Indirectness** | **Imprecision** | **Other considerations** | **glucocorticoids** | **control** | **Relative (95% CI)** | **Absolute (95% CI)** |  |  |
| **Seroma aspiration rate (assessed with: OR)** | | | | | | | | | | | | |
| 4 | randomised trials | serious^a^ | very serious^b^ | not serious | serious^c^ | none | 23/226 (10.2%) | 72/227 (31.7%) | **OR 0.25** (0.02 to 2.77) | **213 fewer per 1,000** (from 308 fewer to 246 more) | ⨁◯◯◯ Very low^a,b,c^ | IMPORTANT |
| **Drainage volume on 1st post-operative day (assessed with: MD)** | | | | | | | | | | | | |
| 4 | randomised trials | not serious | serious^d^ | not serious | very serious^c^ | none | 170 | 173 | - | MD **19.26 ml lower** (488.54 lower to 450.01 higher) | ⨁◯◯◯ Very low^c,d^ | NOT IMPORTANT |
| **Wound necrosis rate (assessed with: RR)** | | | | | | | | | | | | |
| 4 | randomised trials | not serious | not serious | not serious | serious^c^ | none | 4/182 (2.2%) | 11/184 (6.0%) | **RR 0.49** (0.16 to 1.46) | **30 fewer per 1,000** (from 50 fewer to 28 more) | ⨁⨁⨁◯ Moderate^c^ | IMPORTANT |
| **Wound dehiscence rate (assessed with: RR)** | | | | | | | | | | | | |
| 3 | randomised trials | not serious | not serious | not serious | extremely serious^c^ | none | 0/162 (0.0%) | 6/162 (3.7%) | **RR 0.25** (0.01 to 4.27) | **28 fewer per 1,000** (from 37 fewer to 121 more) | ⨁◯◯◯ Very low^c^ | IMPORTANT |

**CI:** confidence interval; **MD:** mean difference; **OR:** odds ratio; **RR:** risk ratio

#### Explanations

a. In most studies included, there were at least some concerns regarding the risk of bias. Therefore, the authors have decided to downgrade the level of evidence.

b. Heterogeneity with the I2 test was 85% (64%-94%), corresponding to a very serious inconsistency. (0-25% low level of heterogeneity = not serious inconsistency, 25-50% moderate level of heterogeneity = serious inconsistency, 50% < high level of heterogeneity = very serious inconsistency).

c. Considering that the 95% CI fails to exclude important benefit or important harm, the authors decided to rate down for imprecision.

d. Heterogeneity with I2 test was 48% (0%-85%), which corresponds to a serious inconsistency. (0-25% low level of heterogeneity = not serious inconsistency, 25-50% moderate level of heterogeneity = serious inconsistency, 50% < high level of heterogeneity = very serious inconsistency).


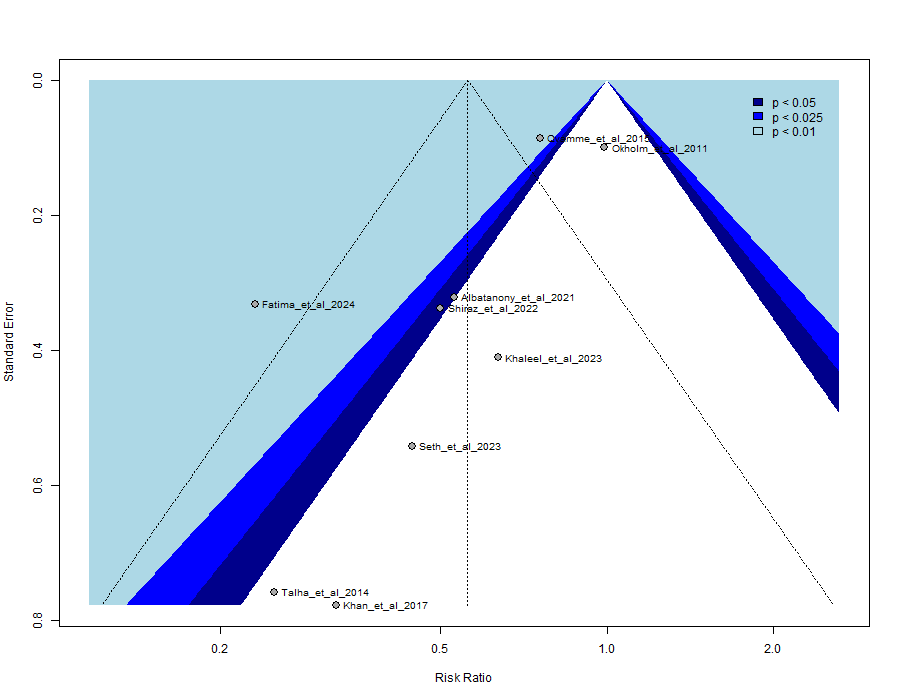


**Figure S18.** Funnel plot for seroma formation rate.


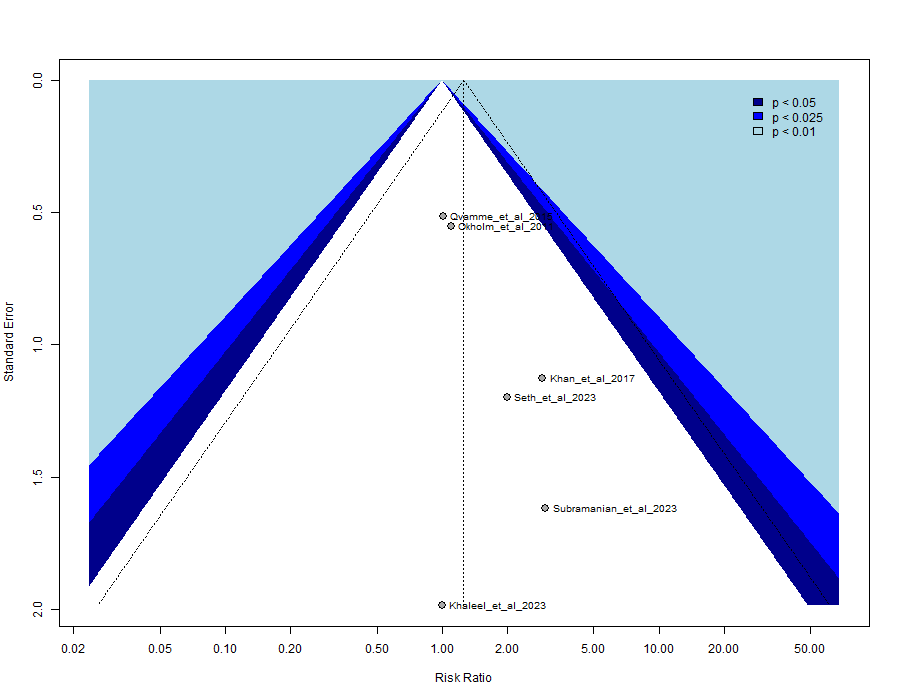


**Figure S19.** Funnel plot for wound infection rate.


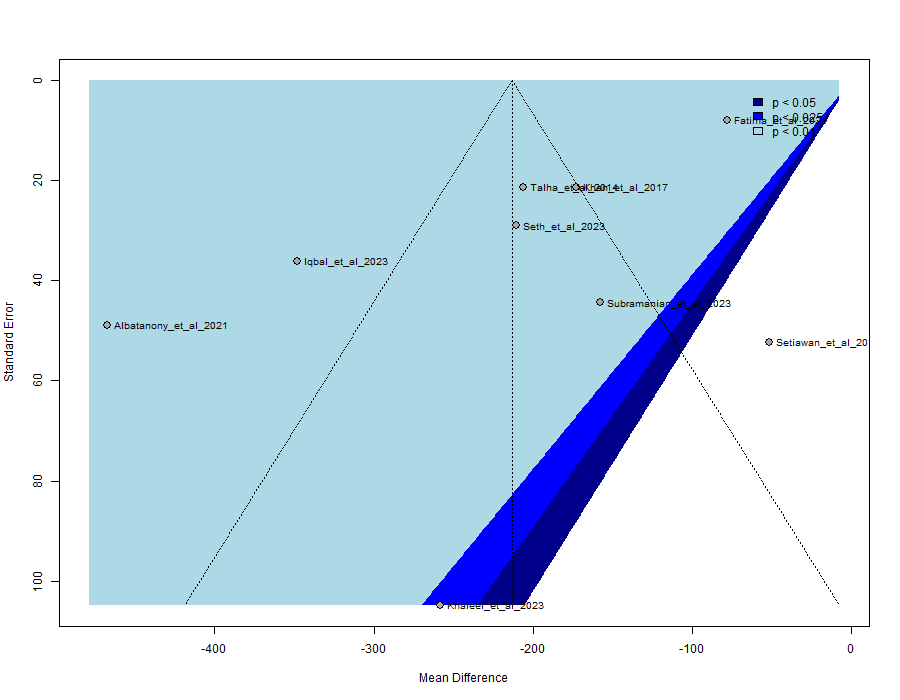


**Figure S20.** Funnel plot for total volume of drainage.


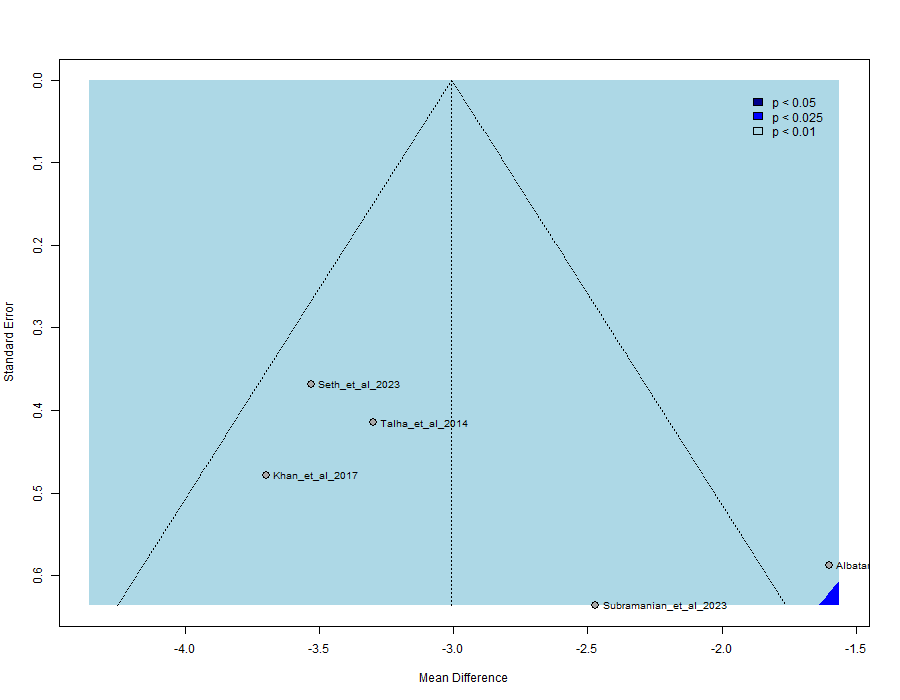


**Figure S21.** Funnel plot for days to drain removal.


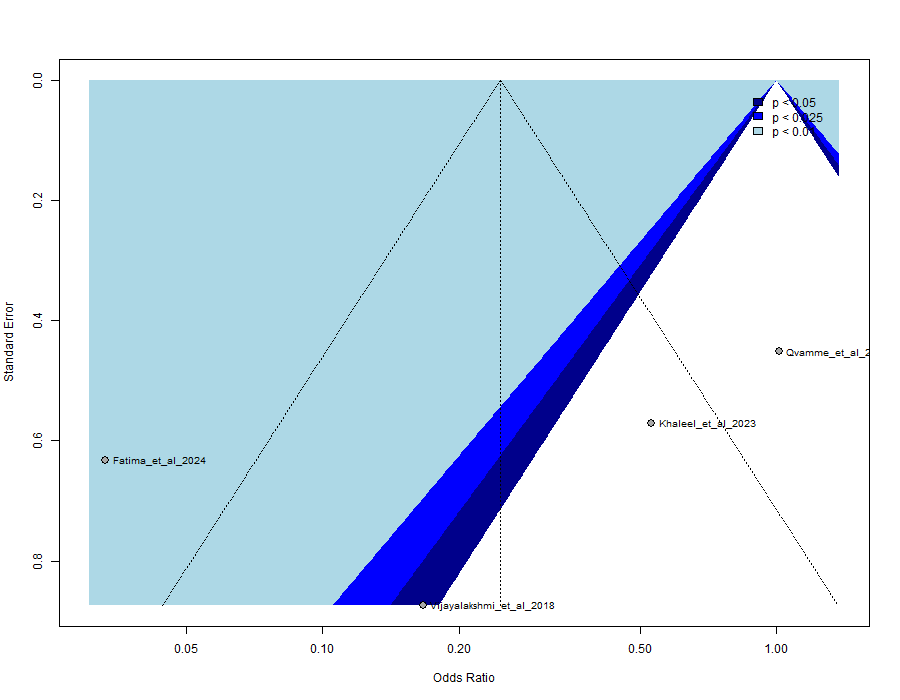


**Figure S22.** Funnel plot for seroma aspiration rate.


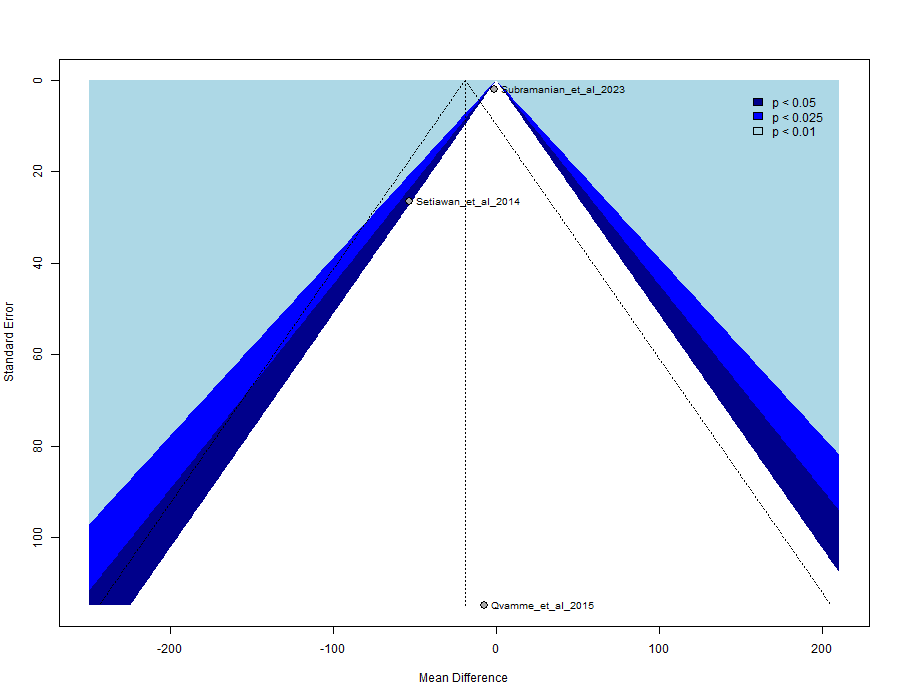


**Figure S23.** Funnel plot for drainage volume on 1^st^ postoperative day.


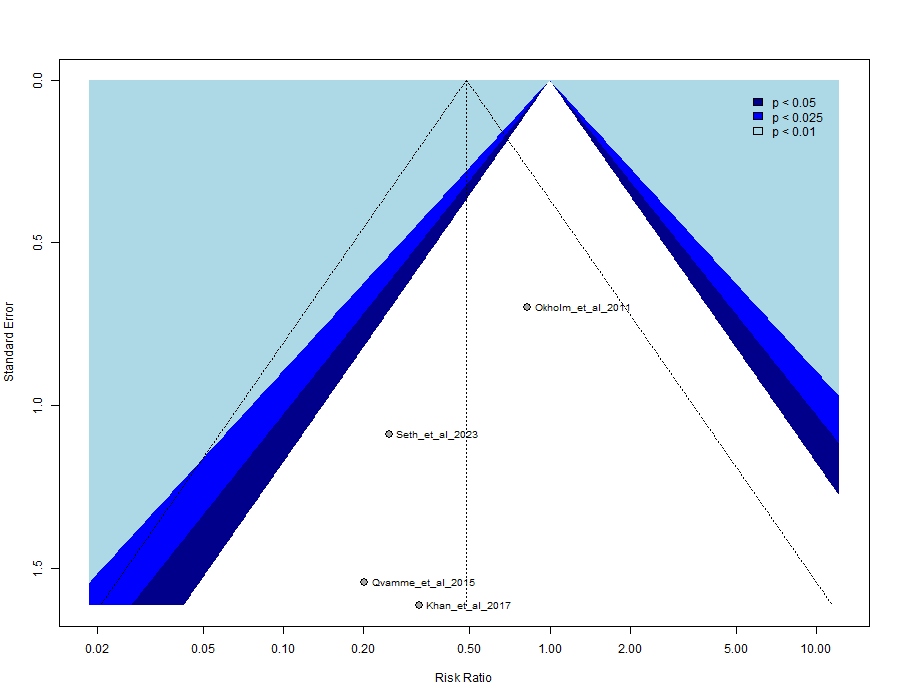


**Figure S24.** Funnel plot for wound necrosis rate.

**
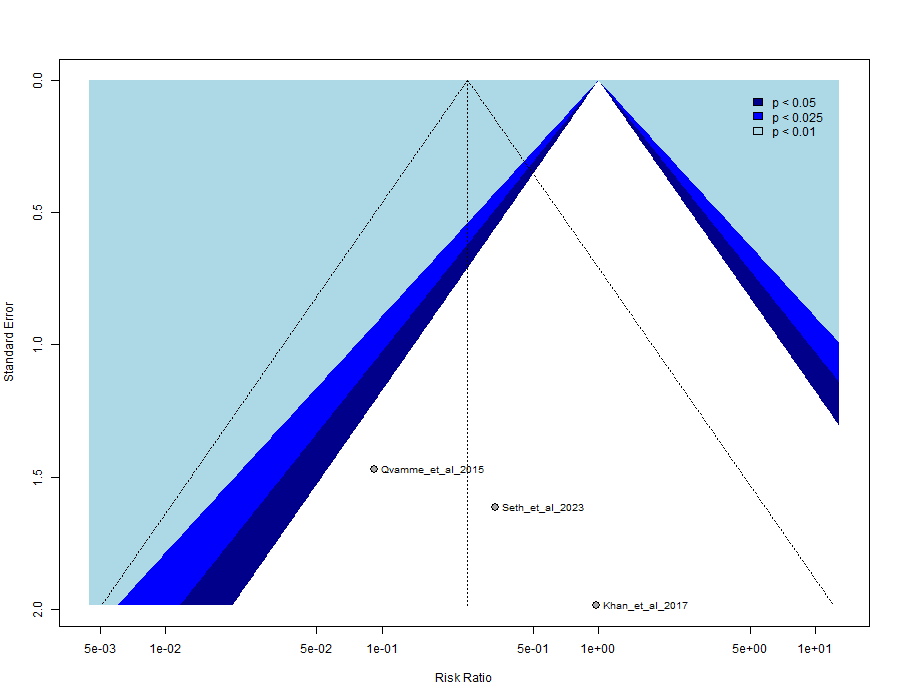
**

**Figure S25.** Funnel plot for wound dehiscence rate.


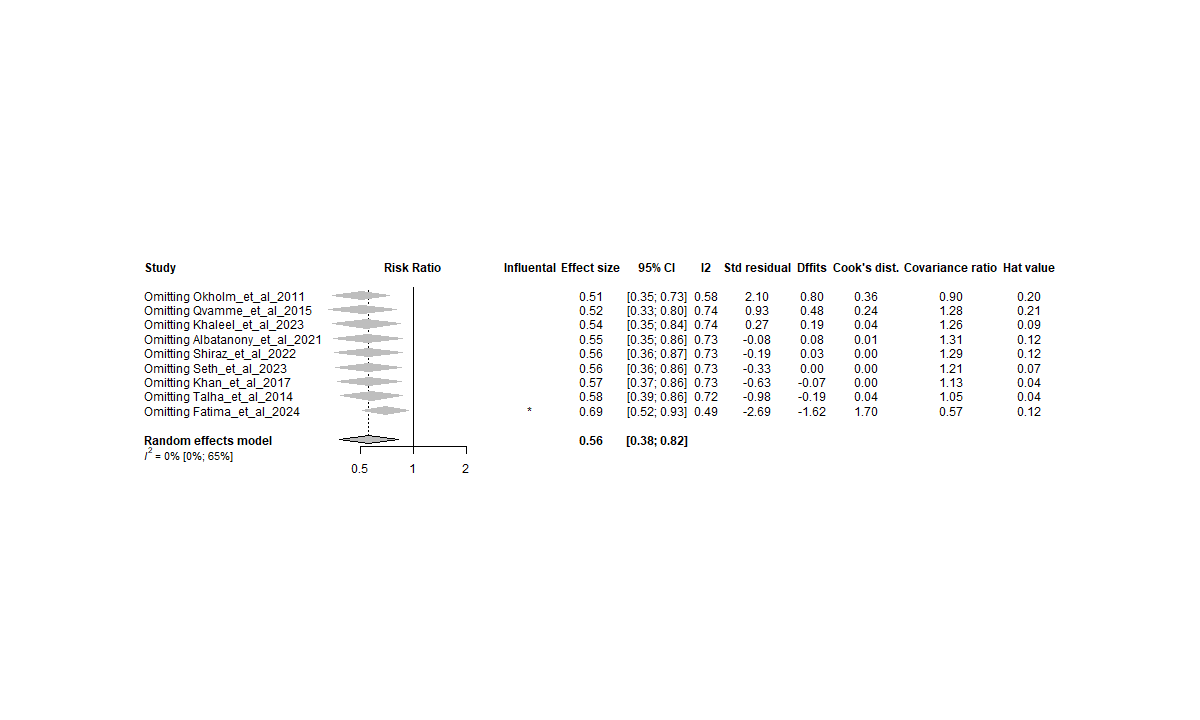


**Figure S26.** Influence forest plot for seroma formation rate. CI, confidence interval.


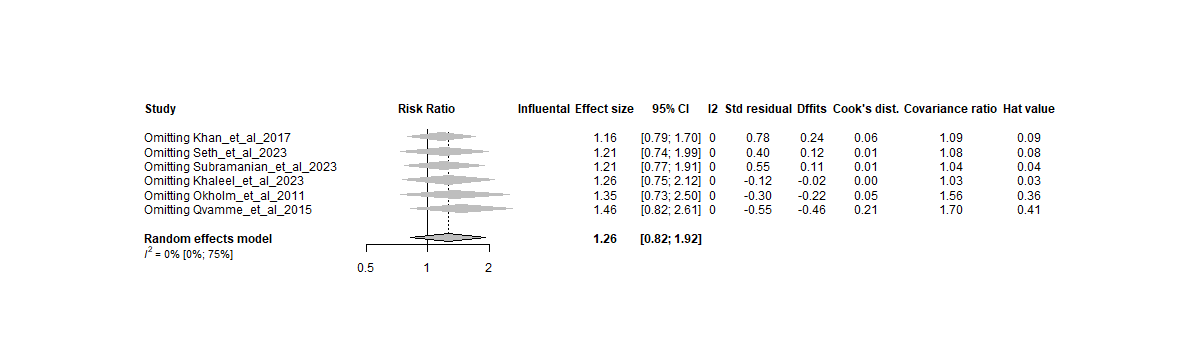


**Figure S27.** Influence forest plot for wound infection rate. CI, confidence interval.


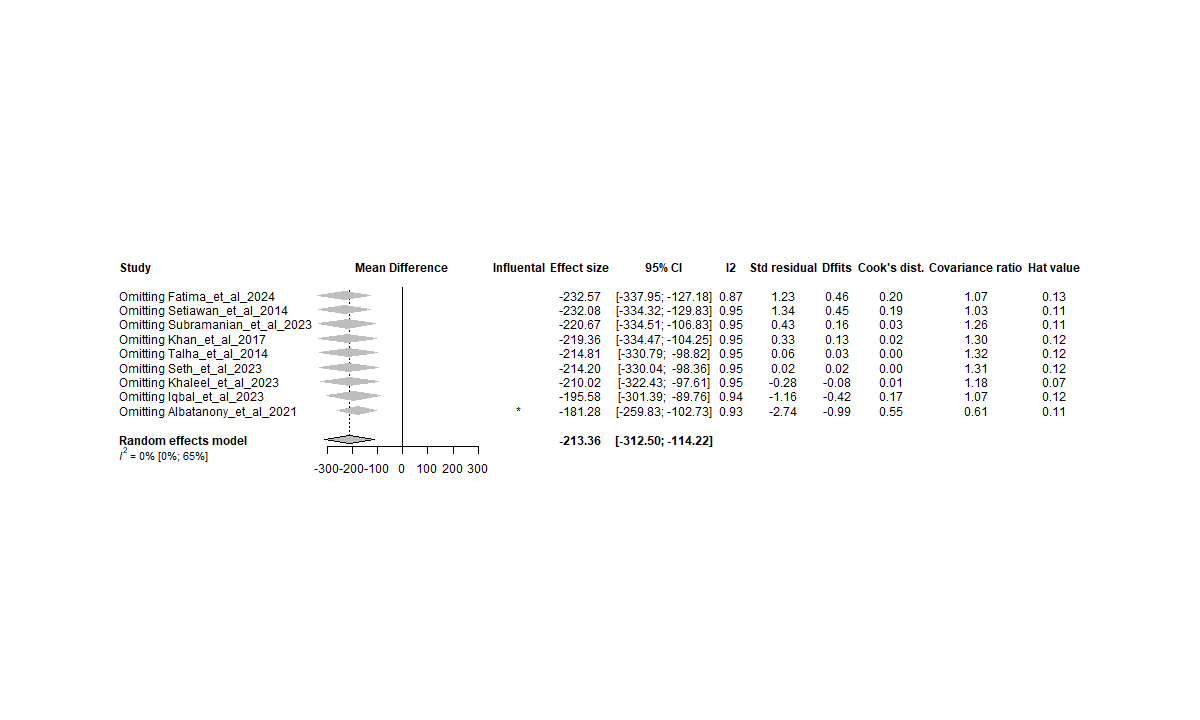


**Figure S28.** Influence forest plot for total volume of drainage. CI, confidence interval.


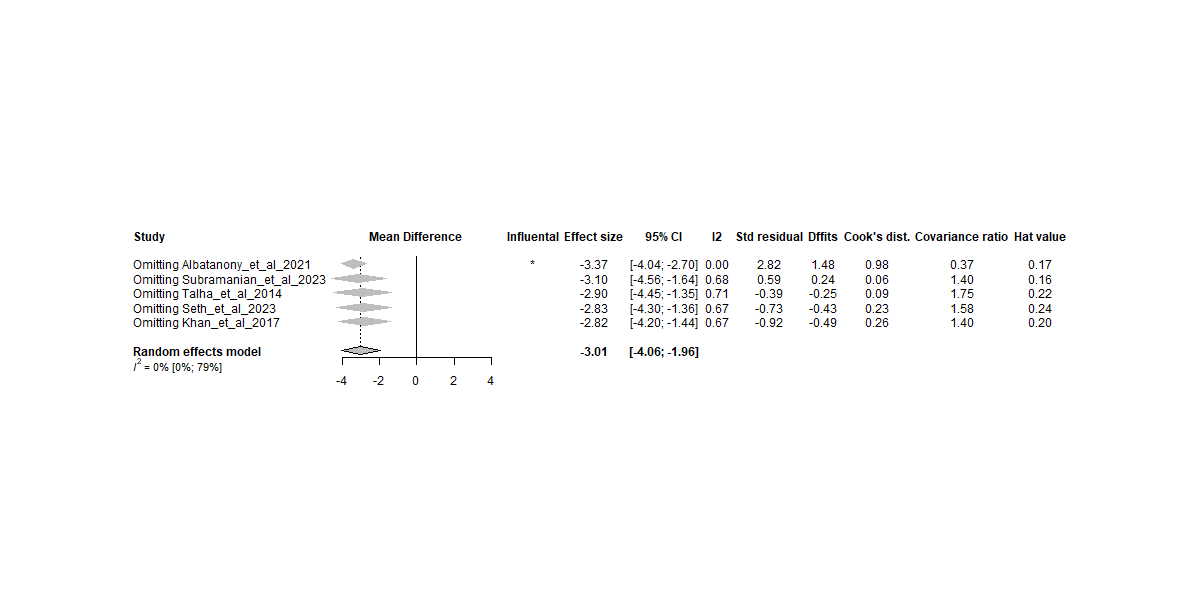


**Figure S29.** Influence forest plot for days to drain removal. CI, confidence interval.


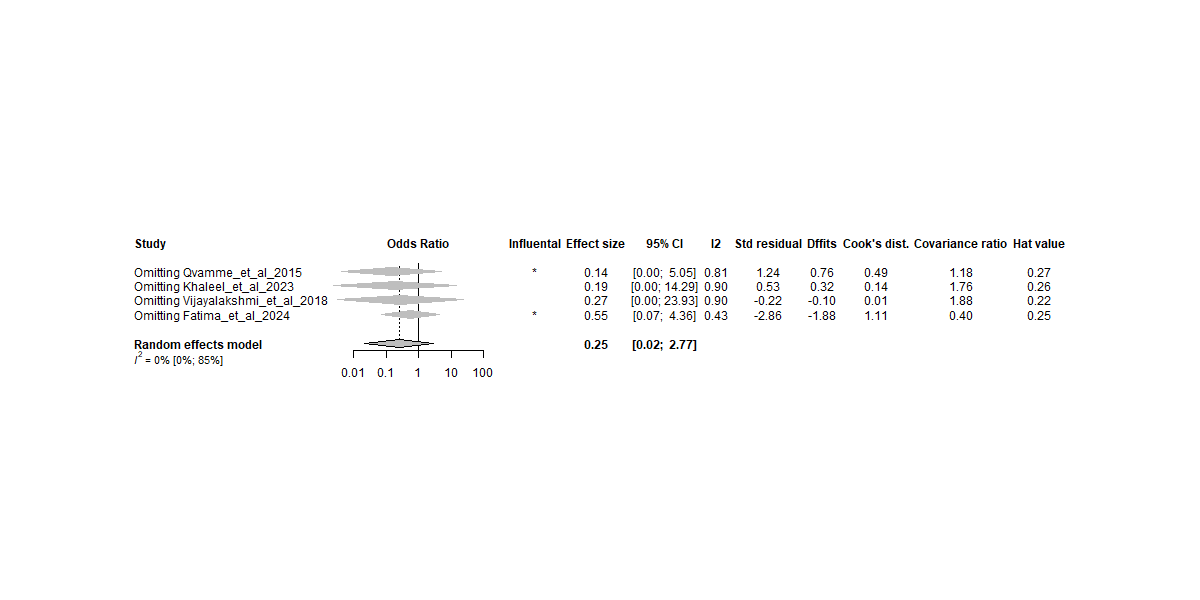


**Figure S30.** Influence forest plot for seroma aspiration rate. CI, confidence interval.


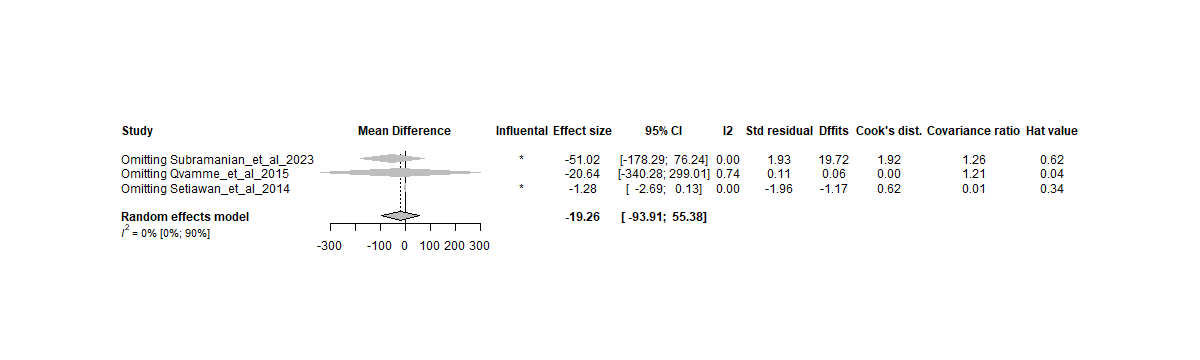


**Figure S31.** Influence forest plot for drainage volume on 1^st^ post-operative day. CI, confidence interval.


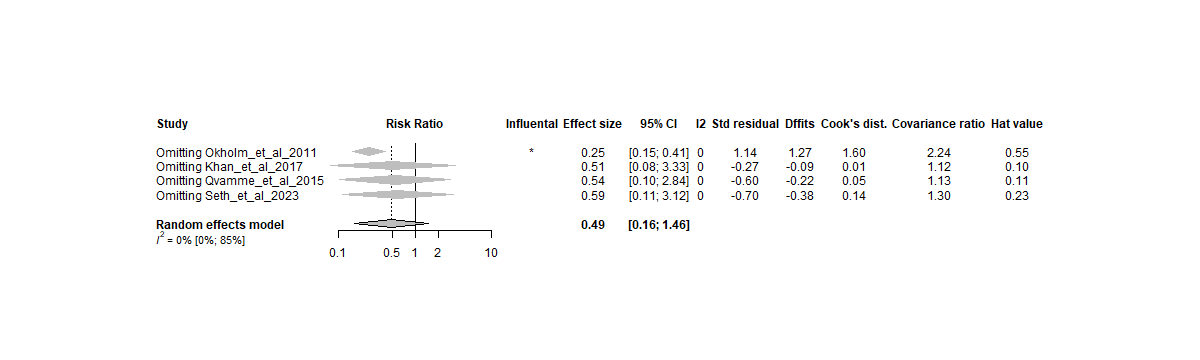


**Figure S32.** Influence forest plot for wound necrosis rate. CI, confidence interval.


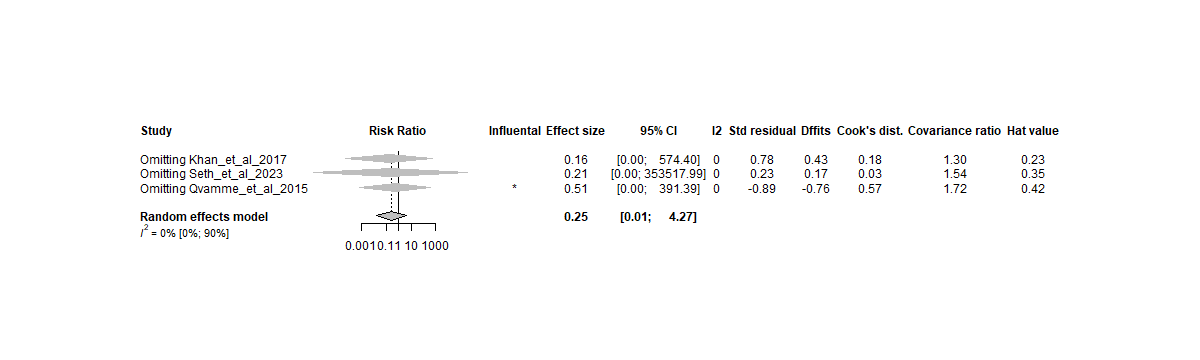


**Figure S33.** Influence forest plot for wound dehiscence rate. CI, confidence interval.

**References**

[1] Fatima S, Shafique MS, Shabana B, Nawaz S, Khan JS, Hasan SW. The Prevention of Seroma Formation Following Modified Radical Mastectomy by Intravenous Hydrocortisone Injection. Cureus 2024;16:e55017.

[2] Khaleel Y, Choudhari A, Abhishek CV. Assessment Of The Total Volume Of Seroma Aspirated And Early Drain Removal Following The Use Of Injection Methylprednisolone In Patients Undergoing Modified Radical Mastectomy. Journal of Cardiovascular Disease Research 2023;14:1117-23.

[3] Qvamme G, Axelsson CK, Lanng C, Mortensen M, Wegeberg B, Okholm M, Arpi MR, Szecsi PB. Randomized clinical trial of prevention of seroma formation after mastectomy by local methylprednisolone injection. Br J Surg 2015;102:1195-203.

[4] Vijayalakshmi S. A comprehensive study on the effect of injection methylprednisolone in post mastectomy seroma. International Archives of Integrated Medicine 2018;5:43.

[5] Setiawan J, Abdurahman M, Rizki KA. Effectiveness of Methylprednisolone on Post-Operative Seroma Formation Following Radical Modified Mastectomy. Majalah Kedokteran Bandung-Mkb-Bandung Medical Journal 2014;46:88-93.

[6] Okholm M, Axelsson CK. No effect of steroids on seroma formation after mastectomy. Dan Med Bull 2011;58:A4241.

[7] Subramanian P, Arumugam M, Vaithianathan R. Role of methylprednisolone in the prevention of seroma formation after mastectomy: A randomized controlled trial. Indian J Cancer 2023;60:206-10.

[8] Seth US, Perveen S, Khan I, Ahmed T, Kamal MT, Khomusi MM. Effect of preoperative intravenous steroids on seroma formation after modified radical mastectomy. J Pak Med Assoc 2023;73:69-73.

[9] Khan MA. Effect Of Preoperative Intravenous Steroids On Seroma Formation After Modified Radical Mastectomy. J Ayub Med Coll Abbottabad 2017;29:207-10.

[10] Albatanony AA, Assar, A.M.A. and El Balshy, M.A.E. Correlation between C-reactive protein, intravenous hydrocortisone, systemic tranexamic acid and post mastectomy seroma. International Surgery Journal 2021;8, 9 (Aug. 2021).

[11] Muhammad Nasir Iqbal AU, Sommayya Aftab, Mansab Ali, Syed Muhammad Mohsin Azeem, Wafa Najeeb. . Effect of Preoperative Steroid Injection on Wound Drainage after Modified Radical Mastectomy. Pakistan Journal of Medical & Health Sciences 2023;17(04).

[12] Shiraz DA, Qazi M, Sabir I, Jameel MK, Aqeel CHM, Muneer M. Methylprednisolone for Prevention of Seroma Formation after Mastectomy. Pakistan Journal of Medical and Health Sciences 2022;16:203-05.

[13] Talha A, Ramadan R, Abdelhamid S, Hamdi S. Postmastectomy seroma: how much is it affected by serum levels of IL-6 and CRP and how much is it reduced by intravenous hydrocortisone injection? Egyptian Journal of Surgery 2015;34:17-21.
